# Three-dimensional geometry controls division symmetry in stem cell colonies

**DOI:** 10.1242/jcs.255018

**Published:** 2021-07-29

**Authors:** Agathe Chaigne, Matthew B. Smith, Rocio Lopez Cavestany, Edouard Hannezo, Kevin J. Chalut, Ewa K. Paluch

**Affiliations:** 1 MRC Laboratory for Molecular Cell Biology, University College London, London WC1E 6BT, UK; 2 IST Austria, 3400 Klosterneuburg, Austria; 3 Wellcome/MRC Stem Cell Institute, University of Cambridge, Cambridge CB2 0AW, UK; 4 Department of Physiology, Development and Neuroscience, University of Cambridge, Cambridge CB2 3DY, UK

**Keywords:** 3D cell division, E-cadherin, Embryonic stem cells, Mitosis, Spindle positioning

## Abstract

Proper control of division orientation and symmetry, largely determined by spindle positioning, is essential to development and homeostasis. Spindle positioning has been extensively studied in cells dividing in two-dimensional (2D) environments and in epithelial tissues, where proteins such as NuMA (also known as NUMA1) orient division along the interphase long axis of the cell. However, little is known about how cells control spindle positioning in three-dimensional (3D) environments, such as early mammalian embryos and a variety of adult tissues. Here, we use mouse embryonic stem cells (ESCs), which grow in 3D colonies, as a model to investigate division in 3D. We observe that, at the periphery of 3D colonies, ESCs display high spindle mobility and divide asymmetrically. Our data suggest that enhanced spindle movements are due to unequal distribution of the cell–cell junction protein E-cadherin between future daughter cells. Interestingly, when cells progress towards differentiation, division becomes more symmetric, with more elongated shapes in metaphase and enhanced cortical NuMA recruitment in anaphase. Altogether, this study suggests that in 3D contexts, the geometry of the cell and its contacts with neighbors control division orientation and symmetry.

This article has an associated First Person interview with the first author of the paper.

## Abstract (precis)

**Summary:** Mouse embryonic stem cells display asymmetric divisions in 3D colonies, with cell–cell junctions and cell geometry acting as key regulators of division symmetry.

## INTRODUCTION

Cell division is central to embryonic development because it allows the increase in cell numbers necessary to build a multicellular organism. The direction along which a cell divides (division orientation), as well as whether or not the division is symmetric in both size and content, can be crucial to cell fate. Indeed, asymmetric divisions lead to the generation of two different daughter cells, a process that is key to development and homeostasis across species. For example, the one-cell stage *Caenorhabditis elegans* embryo divides asymmetrically in size and content, which determines the anterior-posterior axis of the future animal (Gönczy and Rose, 2005). As another example, asymmetric division of neuroblasts in *Drosophila* and *C. elegans* allows the generation of a stem cell and a future differentiated cell (Cabernard and Doe, 2009; Ou et al., 2010). Division orientation is also important to cell fate during mammalian development. In particular, division orientation in 8- and 16-cell mouse embryos has been shown to direct cell positioning, and in turn cell signaling and fate (Korotkevich et al., 2017; Maître et al., 2016; Niwayama et al., 2019).

Notably, in early development, cell division often occurs in a three-dimensional (3D) context, where cells are surrounded by neighbors in all directions. Understanding the control of division orientation and symmetry in 3D is thus crucial for investigating the mechanisms regulating cell fate in development. However, the regulation of the orientation and symmetry of cell division have mostly been studied in isolated cells cultured on two-dimensional (2D) substrates or in cells in other 2D contexts, such as epithelial sheets. In such 2D contexts, division orientation and symmetry depend primarily on the positioning of the mitotic spindle, which relies on cross-talk between the spindle and spindle-positioning proteins at the metaphase cell cortex (McNally, 2013). In many cells types, spindle positioning follows the Hertwig, or ‘long-axis’, rule (Hertwig, 1884), which postulates that cells will divide along their interphase long axis. Cells generally round up in mitosis and how they ‘remember’ the position of their interphase long axis is still under debate, but this appears to rely on a memory of this axis and/or of the forces exerted within and on interphase cells. For example, in HeLa cells, rounded mitotic cells maintain retraction fibers, whose distribution reflects the shape the cell had in interphase, and which control the position of the metaphase spindle (Fink et al., 2011; Théry et al., 2007; Wyatt et al., 2015). Similarly, in the developing *Drosophila* notum epithelium, tricellular junctions, which are positioned according to interphase cell shape before mitotic rounding, control spindle positioning (Bosveld et al., 2016).

The mechanisms ensuring spindle positioning according to interphase cell shape mostly operate in metaphase. At the molecular level, spindle positioning is transduced by the recruitment of a ternary protein complex [comprising the nuclear mitosis apparatus protein (NuMA; also known as NUMA1), Gαi protein 1 (GNAI1), and LGN (also known as G-protein-signaling modulator 2, GPSM2) in mammalian cells] to the cell cortex (the thin actin network that supports the plasma membrane) in metaphase. Upon cortical recruitment, the complex in turn recruits and pulls on the astral microtubules. For example, in the *Drosophila* epithelium, Mud, the *Drosophila* homolog of NuMA, accumulates at tricellular junctions and orients the metaphase spindle (Bosveld et al., 2016). In HeLa cells, after metaphase, NuMA is maintained at the cortex throughout anaphase where it further participates in maintaining the central position of the spindle, thus ensuring division symmetry (Kiyomitsu and Cheeseman, 2013). Little is known about the role of NuMA in anaphase in other systems (Kiyomitsu and Cheeseman, 2013) and the localization of the NuMA–Gαi–LGN complex, and other spindle positioning cues, in cells dividing in 3D environments has received little attention.

Here, we investigate spindle positioning and the symmetry of cell division in a 3D environment using embryonic stem cell (ESC) 3D colonies, a model system for the mammalian inner cell mass at the blastocyst stage. We use automated 3D segmentation to precisely quantify cell size at division exit and find that cells at the colony periphery display strong size asymmetries between daughter cells, whereas cells inside the colony do not. Our data suggest that this is due to a high mobility of the spindle in the cells at the colony surface. We further observe that spindle mobility and division asymmetry correlate with heterogeneous distribution of the cell­–cell junction protein E-cadherin between the two prospective daughter cells. Finally, we show that at the exit from naïve pluripotency, when cells spread and become responsive to lineage differentiation signals, cell division becomes more symmetric, and that, concomitantly, the key regulator of spindle positioning NuMA becomes recruited to the cell cortex at anaphase. Together, our data strongly suggest that the division machinery significantly differs between 2D and 3D environments.

## RESULTS

### ESCs growing at the periphery of 3D colonies display strong size asymmetries at cell division

**Fig. 1.**
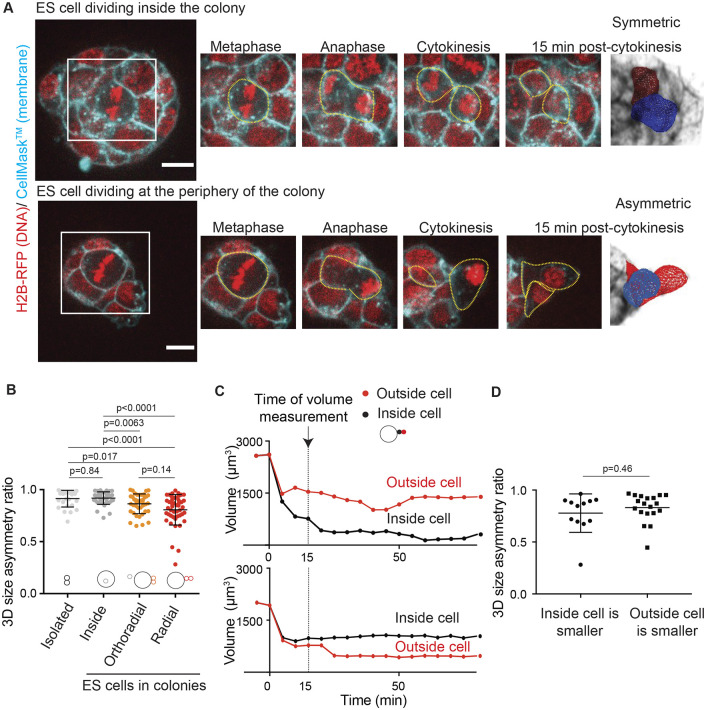
**ESCs display strong size asymmetries at cell division.** (A) Representative time-lapse images of colonies of naïve ESCs expressing H2B–RFP (red) and labeled with CellMask™ Deep Red (cyan) with a cell dividing inside the colony (top) and a cell dividing at the periphery of the colony (bottom). A single *Z*-plane is shown. Scale bars: 10 µm. Cell boundaries are highlighted with yellow dotted line. Images on the right illustrate segmented 3D cell shapes. (B) Dot plot representing the size asymmetry ratio measured in 3D between daughter cells for single ESCs (‘isolated’, light gray), ESCs dividing inside of a colony (‘inside’, medium gray) and ESCs dividing at the periphery of a colony with the mitotic spindle oriented parallel (‘orthoradial’, orange) or perpendicular (‘radial’, red) to the colony border. The mean±s.d. (*N*=4) are shown. Datapoints corresponding to isolated ESCs were replotted from Chaigne et al. (2020), data for cells dividing in colonies were obtained by re-analyzing videos acquired for Chaigne et al. (2020). (C) Example plots showing the evolution of the volumes of daughter cells after cell division at the periphery of the colony, in cases where the outside cell is the bigger one (top) or the smaller one (bottom). 0 is the time of cytokinesis. The time when the size asymmetry reported in D is measured (15 min) is highlighted with a dashed line. (D) Dot plot showing the size asymmetry ratio between daughter cells for cells dividing at the periphery of the colony, with the outside-positioned daughter cell being the bigger one (left) or the smaller one (right). The mean±s.d. are shown (*N*=3). *P*-values were calculated with a Mann–Whitney test.

To investigate spindle positioning and division symmetry in a 3D context, we used mouse ESCs as a model system. When plated on a gelatin substrate in the pluripotency sustaining medium 2i+LIF (Mulas et al., 2019; Ying et al., 2008; see Materials and Methods), ESCs grow in 3D colonies that are usually a few cells thick (Fig. S1A,B, Movies 1–4), and are able to exit naïve pluripotency in a similar manner to the cells in the peri-implantation blastocyst (Kalkan et al., 2017). To track cell division dynamics, we used an ESC line expressing histone 2B (H2B) tagged with RFP (Cannon et al., 2015). We tested the ability of this line to contribute to an embryo by injecting H2B–RFP-expressing ESCs into a blastocyst of an albino C57BL/6 mouse. We observed that the chimeric mouse coat displayed considerable brown patches, showing that the injected cells integrated well into the blastocyst and significantly contributed to the embryo (see Materials and Methods and Fig. S2A). We then labeled cell membranes using CellMask™ and monitored 3D cellular volume throughout cell division, using a custom plugin that we previously developed (Smith et al., 2016). Our previous work has shown that while single isolated ESCs divide relatively symmetrically, ESCs dividing in 3D colonies can display significant size asymmetry between daughter cells (Chaigne et al., 2020). To test the influence of the 3D environment on cell division, we asked whether the level of asymmetry depended on cell position and on the orientation of the division with respect to the colony (Fig. 1A,B). We defined the division asymmetry ratio as the ratio of the volume of the smaller future daughter cell over the volume of the bigger daughter cell 15 min after cytokinesis onset (Fig. S2B).

We found that ESCs where division took place entirely inside the colony divided mostly symmetrically, similar to what was seen for isolated ESCs; however, ESCs dividing at the periphery of colonies often displayed significant size asymmetry between daughter cells (Fig. 1A,B; Movies 5, 6). The proportion of peripheral divisions depended on the colony size, but even large colonies displayed a significant number of cells dividing at the periphery (Fig. S2C). Cells dividing at the periphery of the colonies with the spindle oriented perpendicular to the colony border (‘radial’ division orientation) displayed highest size asymmetries between daughter cells (Fig. 1B). For these cells with radial division orientation, there was no preferential direction of the asymmetry; indeed, the smallest of the two daughter cells had the same probability to be positioned away from or towards the colony center (Fig. 1C,D; Fig. S2D). Together, these data indicate that cell division introduces significant size heterogeneity in mouse ESCs growing in 3D colonies, and that division asymmetries are highest for cells dividing radially on the surface of the colonies.

### Size asymmetries at division are not the result of cortical contractions

We then explored the mechanisms underlying asymmetric division in ESCs. We observed that cells at the colony periphery displayed significant shape instabilities characterized by strong contractions and blebbing (Fig. S3A, Movie 7). Previously, myosin-driven contractions at the cell poles during cytokinesis have been shown to lead to asymmetric division in neuroblasts (Cabernard et al., 2010; Ou et al., 2010). We thus hypothesized that polar surface contractions and instabilities could be responsible for division asymmetries in ESCs. To assess this hypothesis, we first quantified the occurrence of polar shape instabilities in ESCs dividing at different locations in the 3D colonies. Visual assessment of 3D stacks suggested that 57% of dividing cells showed unstable shapes during cytokinesis (Movie 7). To assess shape instabilities in a more unbiased manner, we further analyzed cell curvature dynamics in 2D, focusing on the midplane of each prospective daughter cell (see Materials and Methods). This analysis was consistent with our visual assessment of 3D stacks, with the cells visually classified as unstable displaying significantly more variable contours (Fig. S3B–D). Cells dividing radially displayed more shape instabilities (Fig. S3E), and cells displaying significant shape instabilities also displayed higher division asymmetries (Fig. S3F).

We then asked whether the observed shape instabilities might drive division asymmetry. We noticed that myosin-II accumulated on the outside of ESC colonies (Fig. S3G,H), suggesting that high levels of myosin at the cell poles could cause polar shape instabilities, as previously reported (Cabernard et al., 2010; Sedzinski et al., 2011). We thus interfered with myosin activity to reduce polar contractions and shape instabilities. We treated cells with 1 µM of the myosin-activity inhibitor Blebbistatin, which at such low doses slowed down cytokinesis (Fig. S3I, Movie 8) but did not prevent cell division. Blebbistatin treatment considerably reduced cytokinetic cell shape instabilities (Fig. S3J, Movie 8), but had no strong effect on division asymmetries (measured in 3D, Fig. S3K). Therefore, polar contractions are unlikely to be responsible for division asymmetries in mouse ESC colonies.

### Size asymmetries at division correlate with high spindle mobility in metaphase

**Fig. 2.**
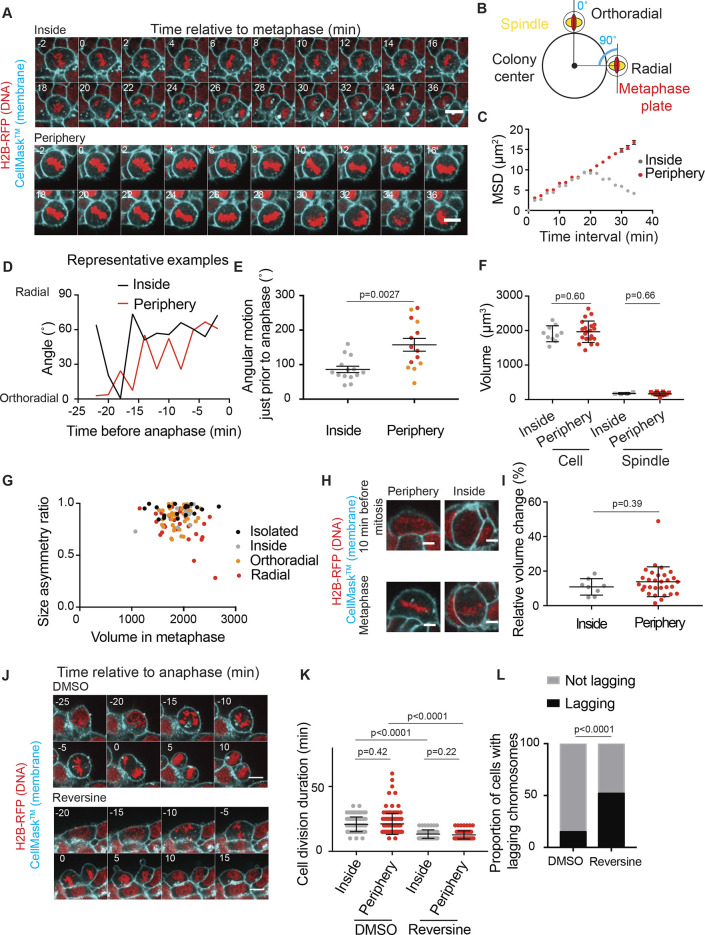
**The spindle is more mobile in cells at the periphery of ESC colonies than in cells dividing inside the colonies.** (A) Representative time-lapse spinning-disk confocal microscopy images of H2B–RFP (red)-expressing naïve ESCs labeled with CellMask™ Deep Red (cyan), with a cell dividing inside the colony (top panels) and a cell dividing at the periphery of the colony (bottom panels). One picture is shown every 2 min. 0 min corresponds to metaphase. One *Z*-plane is shown. Scale bars: 10 µm. (B) Schematic showing a 2D projection of the angle describing the orientation of the metaphase plate (see also panel D; the angle is measured in 3D). (C) Graph showing the 3D mean square displacement of the center of the metaphase plate (see Materials and Methods section) for cells dividing inside (gray) or at the periphery (red) of the colony, as a function of time interval. 0 min represents anaphase. The mean±s.e.m. are shown (*N*=4, *n*=11 inside, *n*=19 at the periphery). (D) Representative example of the dynamics of the angle between the metaphase plate and line connecting the center of the metaphase plate to the center of the colony (see panel B) for a cell dividing inside a colony (black) and a cell dividing at the periphery of the colony (red) as a function of time. 0 min represents anaphase. (E) Dot plot showing the angular motion (the difference in angle between consecutive time points) of the metaphase plate averaged over the last 12 min before anaphase for cells dividing inside the colony (gray) or at the periphery with radial (red) or orthoradial (orange) orientation. The mean±s.e.m. are shown (*N*=3). (F) Dot plot showing the volumes of the cell and of the spindle for cells dividing inside the colony (gray) or at the periphery (red) in H2B–RFP ESCs. The mean±s.d. are shown (*N*=3). (G) Dot plot showing the size asymmetry ratio between daughter cells for ESCs dividing isolated or in 3D colonies (inside or at periphery, as highlighted in legend) as a function of their volume in metaphase. *N*=3. (H) Representative images of H2B–RFP (red) expressing naïve ESCs labelled with CellMask™ Deep Red (cyan) for a cell dividing at the periphery of the colony (left) and a cell dividing inside the colony (right) 10 min before entry into mitosis (defined by nuclear envelope breakdown) (top) and in metaphase (bottom). One Z plane is shown. Scale bars: 10 µm. (I) Dot plot showing the relative volume change before and during mitosis in ESCs dividing inside (left) and at the periphery (right) of the colony. The mean±s.d. are shown. (J) Representative images of H2B–RFP (red)-expressing naïve ESCs labelled with CellMask™ Deep Red (cyan) for a cell treated with DMSO (top) and a cell treated with 100 nM Reversine (bottom). One *Z*-plane is shown. Scale bars: 10 µm. (K) Dot plot showing cell division duration (from nuclear envelope breakdown until anaphase onset) for naïve H2B–RFP ESCs treated with DMSO (left) or 100 nM Reversine (right). Cells dividing inside the colony are plotted in gray, cells dividing at the periphery of the colony are plotted in red. The mean±s.d. are shown *N*=3. (L) Contingency plot showing the percentages of cells dividing with (black) or without (gray) lagging chromosomes after treatment with DMSO (left) or 100 nM Reversine (right). *N*=3. *P*-values were calculated using a Welch's *t*-test (E), Student's *t*-test (F, cell; K, control inside versus Reversine outside), Mann–Whitney test (F, spindle, I,K) and Fisher's test (L).

In order to further investigate how division asymmetries arise in ESCs at the colony surface, we monitored the dynamics of the mitotic spindle, which is key in positioning the cleavage furrow. We used the position and orientation of the mitotic plate in cells expressing H2B–RFP as a proxy for spindle position. We performed fast 3D live imaging and tracking of the metaphase plate in ESCs dividing at the periphery of or inside colonies (Fig. 2A,B; Movies 9, 10). To quantify spindle dynamics, we calculated the 3D mean squared displacement (MSD, a measure of how much the metaphase plate moves during increasing time intervals) of the position of the center of the metaphase plate in the reference frame of the cell, with respect to the final position of the metaphase plate at anaphase. Metaphase plate position displayed extensive fluctuations in 3D, particularly in cells dividing at the periphery of the colony. Indeed, metaphase plates for cells dividing at the periphery of the colony displayed a linear increase of the MSD in time, consistent with an unconstrained diffusion of the spindle, whereas metaphase plates inside colonies showed a plateau or even a decrease of the MSD with increasing time interval, consistent with constrained spindle diffusion (Fig. 2C). This supports the possibility of a less tight regulation of spindle position for cells dividing at the colony periphery. We then quantified metaphase plate angular dynamics in 3D in the reference frame of the colony (Fig. 2D,E). Interestingly, we observed that the metaphase plate also displayed extensive rotations, which significantly decreased in the 10–15 min prior to anaphase in cells dividing inside colonies, but not in cells dividing at the colony periphery (Fig. 2D,E). We did not notice any difference in spindle radial motility between cells dividing radially and orthoradially (Fig. 2E). Differences in cell or spindle volumes could lead to differences in available space for movement, which could in turn affect spindle dynamics. However, we found no significant difference in either cell or spindle volumes between cells dividing inside colonies or at the periphery of colonies (Fig. 2F), and the asymmetry ratio between daughter cells showed no correlation with cell volume in metaphase (Fig. 2G). Furthermore, we verified that, as described in other cell types (Cadart et al., 2014; Son et al., 2015; Zlotek-Zlotkiewicz et al., 2015), ESCs display volume swelling at the beginning of cell division, and observed that the relative volume change was comparable in cells dividing inside or at the periphery of the colony (Fig. 2H,I). Finally, we tested that the increased mobility of the spindle in cells dividing peripherally was not simply due to enhanced division duration or delays in the satisfaction of the spindle assembly checkpoint (SAC). We measured division duration (from nuclear envelope breakdown to anaphase onset) and found no significant difference between cells dividing inside or at the periphery of colonies (Fig. 2J,K, DMSO). We then treated cells with 100 nM of the SAC inhibitor Reversine. As expected, Reversine treatment lead to an increase in the number of cells dividing with lagging chromosomes (Fig. 2J,L) and an overall shortening of division duration (Fig. 2K). However, we found no difference in division duration between Reversine-treated cells dividing inside and at the periphery of colonies (Fig. 2J,K), suggesting that SAC-independent phases of cell division proceed with similar dynamics in the two configurations. Together, these experiments strongly suggest that there is no delay in SAC activation in cells dividing peripherally. Altogether, these results show that division asymmetry in ESCs correlates with high spindle mobility and suggest that enhanced spindle mobility at the colony periphery is not simply caused by differences in cell geometry, division duration or SAC satisfaction.

### Size asymmetries at division correlate with asymmetrically distributed E-cadherin cell–cell contacts

**Fig. 3.**
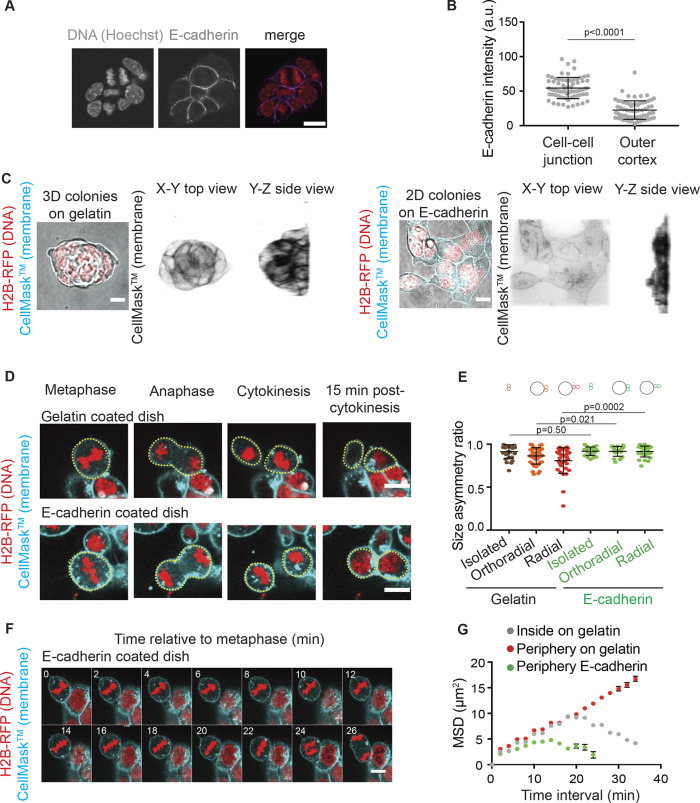
**E-cadherin heterogeneities correlate with size asymmetries at division in ESCs.** (A) Representative confocal images of ESCs stained for E-cadherin and DNA. Scale bar: 10 µm. (B) Dot plot showing the intensity levels of E-cadherin staining in H2B–RFP ESCs, at cell–cell junctions and at cell borders exposed to the outside of the colony (outer cortex). The mean±s.d. are shown (*N*=2). (C) Representative images of colonies of ESCs expressing H2B–RFP (red) and labeled with CellMask™ Deep Red (cyan) on a gelatin-coated substrate (left), and an E-cadherin-coated substrate (right). A *Z*-projection overlaid on a transmitted light image is shown on the left and 3D renditions of a top view (*XY*, middle) and a side view (*YZ*, right) are shown on the right. Scale bars: 10 µm. (D) Representative time-lapse images of dividing ESCs expressing H2B–RFP (red) and labeled with CellMask™ Deep Red (cyan) on a gelatin-coated substrate (top) and on an E-cadherin-coated substrate (bottom). One *Z*-plane is shown. Cell boundaries are highlighted with yellow dotted line. Scale bars: 10 µm. (E) Dot plot representing the size asymmetry ratio between daughter cells at division for ESCs plated on E-cadherin and dividing as single cells (‘isolated’) or at the periphery of a colony with the spindle oriented orthoradially or radially to the colony border. The control data for cells on gelatin from Fig. 1C,D is plotted for reference. The mean±s.d. are shown (*N*=2). (F) Representative time-lapse spinning-disk confocal microscopy images of a H2B–RFP (red) expressing ESC colony labeled with CellMask™ Deep Red (cyan) plated on E-cadherin with one cell dividing at the periphery of the colony. 0 min corresponds to metaphase. One *Z* plane is shown. Scale bar: 10 µm. (G) Graph showing the 3D mean square displacement of the center of the metaphase plate (see Materials and Methods section) for ESCs plated on E-cadherin and dividing at the periphery of the colony (green) as a function of time interval. The mean±s.e.m. are shown (*N*=2, *n*=15). The data corresponding to cells on gelatin from Fig. 2C are plotted in gray and red for reference. 0 min represents anaphase. *P*-values were calculated with a Mann–Whitney test (B,E) or Student's *t*-test (E, radial). a.u., arbitrary units.

We then hypothesized, based on our observation that asymmetric divisions particularly arise at the periphery of colonies (Fig. 1B), that asymmetries in E-cadherin distribution between prospective daughter cells could be responsible for division asymmetries. Indeed, E-cadherin accumulates at cell–cell junctions and therefore is more uniformly localized around cells inside colonies than around cells at the colony surface (Fig. 3A,B; Fig. S4A,B). We thus plated cells on E-cadherin-coated substrates, which lead to naïve ESCs spreading in 2D colonies (Fig. 3C; Movies 11, 12). Thus, as cell division is oriented parallel to the substrate in 2D colonies, both daughter cells are exposed to comparable levels of E-cadherin throughout division, through contact of their bottom surface with the substrate. We first verified that plating ESCs on E-cadherin abolished the difference in E-cadherin cell–cell junction heterogeneities between inner and outer cells; in fact, cells plated on E-cadherin displayed barely any E-cadherin at cell–cell junctions, likely because most of their E-cadherins were engaged with the substrate (Fig. S4A,B). We then assessed division asymmetries on E-cadherin substrates. Cells plated on E-cadherin divided much more symmetrically than cells in 3D colonies (Fig. 3D,E; Movie 12). Furthermore, cells at the periphery of ESC colonies on E-cadherin displayed spindles as stable as cells inside 3D colonies (Fig. 3F,G, green dots, ‘periphery E-cadherin’; Movie 12). Together, these observations suggest that the high spindle mobility and division asymmetry observed at the periphery of 3D ESC colonies could be mediated by unequal E-cadherin distribution between prospective daughter cells.

Since the geometry of the colony was affected when cells were plated on E-cadherin, we sought to verify whether colony spreading could by itself lead to reduced division asymmetries. To do so, we used laminin-coated substrates where ESC colonies adopt spread morphologies similar to cells on E-cadherin (Fig. S5A, Movies 13, 14). We found that, on laminin, ESCs dividing at colony peripheries displayed asymmetries comparable to those for cells dividing at the periphery of 3D colonies, and higher than in similarly positioned cells dividing on E-cadherin (Fig. S5B,C, Movie 14). This suggests that higher cell division symmetry in ESCs plated on E-cadherin is not simply due to the spreading of the colonies. We verified that cells plated on laminin displayed a similar heterogeneity in E-cadherin intensity between inner and outer junctions to cells plated on gelatin (Fig. S4A,B).

Reduced division asymmetries on E-cadherin were also not due to smaller cell volumes, which might confine spindle motion, as cells plated on E-cadherin had volumes comparable to those of cells in 3D colonies (Fig. S5D). Furthermore, cell division duration for cells on E-cadherin substrates was shorter than in 3D colonies, mostly due to a shorter time spent in prometaphase and metaphase (Fig. S5E–H). This suggests that division asymmetries are not the result of longer times spent in the phases of division during which the spindle is positioned. Altogether, these results suggest that inhomogeneity in E-cadherin distribution between the prospective daughter cells during cell division at the periphery of 3D colonies may lead to instabilities in spindle position and strong asymmetries in cell size at cell division.

### Cell division symmetry increases during exit from naïve pluripotency

**Fig. 4.**
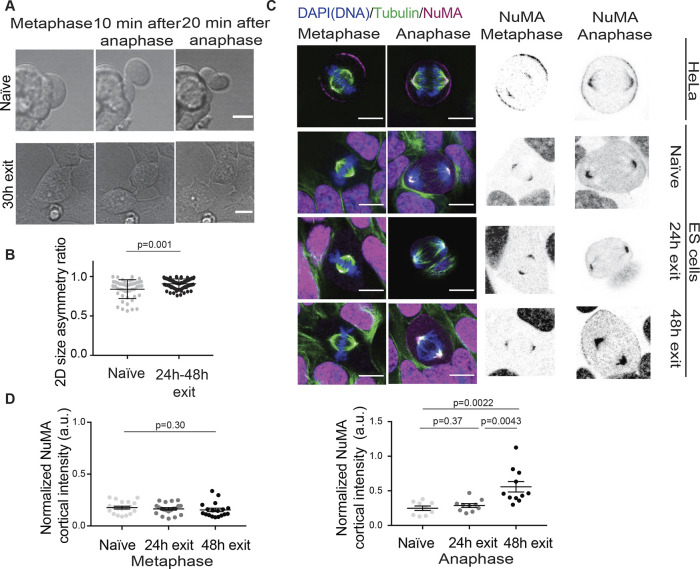
**Division symmetry increases and NuMA becomes recruited to the cortex in anaphase during exit from naïve pluripotency.** (A) Representative transmitted light images of naïve ESCs (top) and ESCs exiting naïve pluripotency (bottom) during cell division. One *Z*-plane is shown. Scale bars: 10 µm. (B) Dot plot showing the 2D size asymmetry ratio between daughter cells measured 20 min after cytokinesis for naïve ESCs (top) and ESCs exiting naïve pluripotency. The mean±s.d. are shown (*N*=2). (C) Left, representative confocal images of HeLa cells (top), naïve ESCs (second row), and cells allowed to exit naïve pluripotency for 24 h (third row) and 48 h (bottom) stained for DNA (blue), α-tubulin (green) and NuMA (magenta). One *Z*-plane is shown. Scale bars: 10 µm. Right, NuMA staining (inverted contrast) in metaphase and anaphase. (D) Dot plots showing the normalized levels of NuMA intensity at the polar cortex in metaphase (left panel) or anaphase (right) for naïve ESCs (light gray), cells exiting naïve pluripotency for 24 h (gray) or 48 h (black). Intensities were normalized to the mean intensity at the spindle poles. The mean±s.e.m. are shown (*N*=3). *P*-values were calculated with a Mann–Whitney test (B), Kruskal–Wallis test (D, metaphase) and Student's *t*-test (D, anaphase) with Welch's correction (D, anaphase, naïve versus 48 h). a.u., arbitrary units.

We then sought to examine whether the levels of division asymmetry are maintained during exit from naïve pluripotency. We induced exit by removing 2i+LIF from the culture medium and assessed division symmetry after 24 h and 48 h. After 24 h, the population should be a mixed population of exited and naïve cells, and most cells will have exited naïve pluripotency at 48 h (Kalkan et al., 2017). When ESCs exit naïve pluripotency, they spread on the substrate (De Belly et al., 2021) (Fig. 4A), making 3D volume measurements from confocal stacks inaccurate in the thinner portions of the cell. Therefore, we used a 2D quantification of cell area as a read-out of cell size. We observed that cells exiting naïve pluripotency displayed significantly more symmetric divisions compared to their naïve counterparts (Fig. 4A,B).

### The spindle positioning protein NuMA becomes enriched at the anaphase cortex during exit from naïve pluripotency

To explore the molecular basis for this increase in division symmetry, we focused on the spindle positioning regulator NuMA. Indeed, NuMA can act to specify spindle positioning as part of the NuMA–Gαi–LGN complex, but also independently of the complex, through interactions with the proteins 4.1G and 4.1R (also known as EPB41L2 and EPB41, respectively) (Kiyomitsu and Cheeseman, 2013). NuMA, Gαi, LGN and 4.1R are all expressed in naïve ESCs (Kalkan et al., 2017; Yang et al., 2019). Therefore, we explored NuMA localization as a good proxy for the localization of spindle positioning complexes. We first verified that as expected, in metaphase and anaphase HeLa cells, NuMA accumulated at the spindle poles, where it is known to organize microtubules, and at the cell cortex (Kiyomitsu and Cheeseman, 2013) (Fig. 4C). To quantify NuMA recruitment to the cortex, while accounting for cell-to-cell staining variation, we quantified the cortical NuMA signal as the ratio between NuMA mean intensity at the polar cortex and mean spindle pole intensity. Strikingly, we observed that while HeLa cells recruit NuMA to the cortex in metaphase, where it contributes to the control of spindle positioning, as previously reported (Kotak et al., 2012; Woodard et al., 2010), naïve ESCs or cells in early stages of naïve pluripotency exit (24 h) did not (Fig. 4C,D). We also observed little cortical enrichment of NuMA in anaphase in naïve ESCs and in cells 24 h after triggering exit from naïve pluripotency. However, NuMA became strongly recruited to the anaphase polar cortex in cells at late stages of exit from naïve pluripotency (48 h) (Fig. 4C,D). Together, this data suggests that enhanced division symmetry upon exit from naïve pluripotency could be mediated by NuMA recruitment to the anaphase cortex. Interestingly, all three components of the NuMA–Gαi–LGN complex are expressed in naïve cells and during exit from naïve pluripotency (Kalkan et al., 2017; Yang et al., 2019). In particular, *NuMA* expression levels are maintained during exit from naïve pluripotency (Kalkan et al., 2017; Yang et al., 2019) suggesting that the low levels of cortical NuMA in naïve cells that we observe are not due to the absence of the protein but to regulation of its localization.

### Enhanced division symmetry is accompanied by NuMA recruitment in anaphase and elongated metaphase cell shapes

**Fig. 5.**
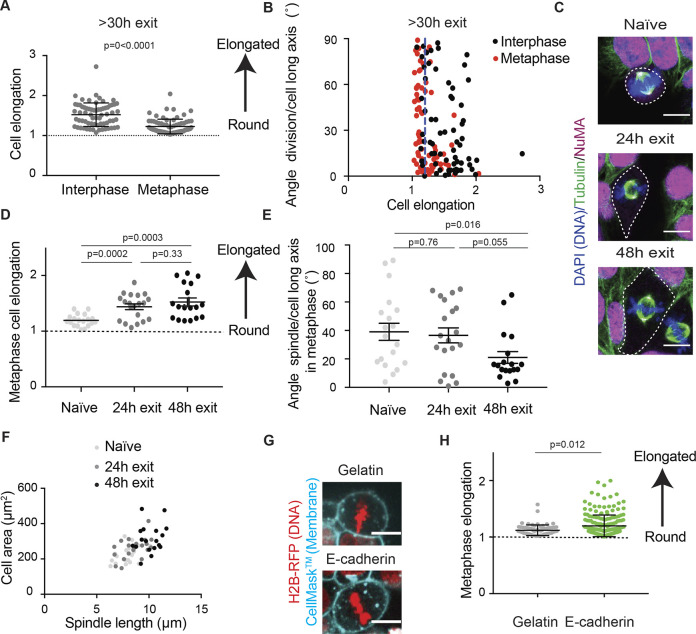
**Cells exiting naïve pluripotency exhibit elongated cell shapes in metaphase.** (A) Dot plot showing the cell elongation in interphase and metaphase for cells 30 to 55 h after induction of exit from naïve pluripotency, measured in live cells. (B) Dot plot showing the angle between the cell long axis in interphase, or in mitosis, respectively, and the final division axis, as a function of cell elongation in interphase (black), or metaphase (red), respectively, measured in live cells 30 to 55 h after induction of exit from naïve pluripotency. The dashed blue line highlights cell elongation of 1.2. (C) Representative confocal images of naïve ESCs (left), and cells allowed to exit naïve pluripotency for 24 h (middle) and 48 h (right) stained for DNA (blue), α-Tubulin (green) and NuMA (magenta) with cell shape outlined (dashed white line). One *Z*-plane is shown. Scale bars: 10 µm. (D) Dot plot showing cell elongation at metaphase for naïve ESCs (light gray), ESCs exiting naïve pluripotency for 24 h (gray) or 48 h (black), measured in fixed cells. The mean±s.e.m. are shown (*N*=3). Of note, the absolute values of cell elongation are different in B and D because the measurements were made in B in live cells between 30 and 55 h after triggering naïve pluripotency exit, and in D in fixed cells at specific time points during exit. (E) Dot plot showing the angle between the spindle and the metaphase long axis for naïve ESCs (light gray), and cells exiting naïve pluripotency for 24 h (gray) or 48 h (black), measured in fixed cells. The mean±s.e.m. are shown (*N*=3). (F) Dot plot showing the cell area as a function of spindle length for naïve ESCs (light gray), and cells exiting naïve pluripotency for 24 h (gray) or 48 h (black). *N*=3. (G) Representative confocal images of a naïve ESC in metaphase in a 3D colony (top) and a naïve ESC in metaphase plated on E-cadherin (bottom)-expressing H2B–RFP (red) cells and labeled with CellMask™ Deep Red (cyan). One *Z*-plane is shown. Scale bars: 10 µm. (H) Dot plot showing the cell elongation in metaphase for naïve ESCs in 3D colonies (gray) or plated on E-cadherin (green). The mean±s.e.m. are shown (*N*=2). *P*-values were calculated with a Mann–Whitney test (A,E,H), Student's *t*-test (D, 24 h versus 48 h; E, naïve versus 24 h) and *t*-test with Welch's correction (D).

In order to explore what might control the cortical recruitment of NuMA in anaphase in cells exiting naïve pluripotency, we characterized cellular shape in these cells. Indeed, interphase cell shape has been proposed to direct metaphase NuMA localization and subsequent division orientation in various cell types (Bosveld et al., 2016; Kiyomitsu and Cheeseman, 2013). We thus measured interphase and metaphase cell elongation in cells >30 h after induction of naïve pluripotency exit and asked whether it correlated with the angle of cell division. We found that while interphase shape was more elongated than metaphase shape (as expected since cells round up for mitosis, Fig. 5A), the cells did not divide along their interphase long axis (Fig. 5B, black dots; the angle between the division axis and the cell long axis was comparable for cells displaying an elongation >1.2 – 82% of the interphase cells – and for cells displaying lower elongation, *P*=0.34). In contrast, we found that for cells that displayed an elongated cell shape in metaphase (cell elongation >1.2, 42% of the mitotic cells, red dots to the right of blue dashed line on Fig. 5B), the division axis was generally within 30° of the metaphase long axis, and the angle between the metaphase long axis and the division axis was significantly smaller for cells displaying an elongation >1.2 than for less-elongated cells (mean 22° compared to 38°, *P*=0.0037). This suggests that, in cells exiting naïve pluripotency, spindle position may correlate better with metaphase cell shape than interphase cell shape.

We thus asked whether, in cells exiting naïve pluripotency, where NuMA is only recruited to the cortex in anaphase, this recruitment correlated with elongated cell shapes in metaphase. We noticed that while naïve ESCs, which display strong division asymmetries and do not recruit NuMA to the cortex in anaphase, displayed round shapes in metaphase; metaphase cell elongation then increased during exit from naïve pluripotency (Fig. 5C,D). Furthermore, the spindle displayed significant alignment along the metaphase long axis at late stages of exit, when metaphase cell elongation was strongest (Fig. 5D,E, 48 h). Spindle orientation along the cell long axis in metaphase was not due to variations in cell size or spindle size, as cell and spindle size scaled at every stage of exit from naïve pluripotency (Fig. 5F). Our data show that recruitment of NuMA to the anaphase cortex correlates with increased cell shape elongation in metaphase and spindle orientation along the metaphase long axis.

NuMA recruitment to the cortex in anaphase is important for controlling division symmetry in HeLa cells (Kiyomitsu and Cheeseman, 2013). In cells exiting naïve pluripotency, NuMA recruitment in anaphase correlated with increased division symmetry and with more-elongated metaphase cell shapes. Interestingly, we also noticed that when naïve ESCs were plated on E-cadherin, where cells divide more symmetrically than in colonies, they exhibited elongated shapes in metaphase (Fig. 5G,H). Finally, the localization of E-cadherin itself remained unchanged through exit from naïve pluripotency on all substrates (Fig. S5A,B), suggesting that the increase in division symmetry at exit from naïve pluripotency is not due to changes in E-cadherin localization. Altogether, these data suggest that metaphase cell shape elongation, which is higher in ESCs plated on E-cadherin and cells exiting naïve pluripotency than in ESCs in 3D colonies, might influence spindle positioning and division symmetry.

## DISCUSSION

During early development and homeostasis, robust cell organization relies on a tight control of cell division orientation. While many studies have investigated the control of division orientation and the mechanisms of spindle positioning in isolated cells or epithelia (Anastasiou et al., 2020; Bosveld et al., 2016; Fink et al., 2011; Hart et al., 2017; Nestor-Bergmann et al., 2014; Théry and Bornens, 2006; Théry et al., 2005, 2007; Wyatt et al., 2015), not much is known about the mechanisms controlling spindle orientation and relative daughter cell sizes in cells growing in disordered 3D environments, such as the early mammalian embryo.

Here, we investigate cell division orientation and symmetry in mouse ESC 3D colonies. We observe that ESCs display strong size asymmetries between daughter cells, especially for cells dividing at the periphery of the colony (Figs 1 and 2). Division asymmetry appears to be due to heterogeneous distribution of E-cadherin at the time of division. In particular, when both daughter cells are in contact with E-cadherin, either through cell–cell contacts when dividing inside colonies or through substrate contact when dividing on E-cadherin, the spindle is more stably positioned at the center of the cell and division is more symmetric (Fig. 3). However, for cells where only one prospective daughter cell displays substantial contact with the colony, the spindle displays high mobility and divisions are asymmetric in size (Figs 1–3). E-cadherin has been implicated in orienting cell division along the epithelial plane in several mammalian epithelia (den Elzen et al., 2009; Lázaro-Diéguez and Müsch, 2017; Lough et al., 2019). For instance, in the skin epidermis, cell–cell adhesions have been shown to play a role in refining the position of the spindle at the end of mitosis, independently of the spindle positioning protein LGN (Lough et al., 2019), while E-cadherin directs spindle orientation through LGN in prostate epithelia (Wang et al., 2018). It will be interesting in the future to investigate the mechanistic link between spindle positioning and E-cadherin in ESCs. Interestingly, E-cadherin also plays a role in maintaining naïve pluripotency of mouse ESCs (Soncin and Ward, 2011; Soncin et al., 2009), and the role of E-cadherin in signaling is ancestral to its role in adhesion (Salinas-Saavedra et al., 2019 preprint). It is tempting to speculate that the strong asymmetries observed during naïve ESC divisions could be a by-product of E-cadherin's importance for pluripotency.

Intriguingly, our recent study showed that the strong division asymmetries displayed by ESCs do not appear to affect the dynamics of naïve pluripotency exit (Chaigne et al., 2020). Instead, we showed that even though cells exit naïve pluripotency after a cell division, sister cells display similar pluripotency exit dynamics irrespective of the level of asymmetry of cell division. Thus, division asymmetries do not appear to affect cell fate during early differentiation directly (Chaigne et al., 2020). Another role of asymmetric divisions resulting from E-cadherin inhomogeneities in ESC colonies could be to introduce noise in the population at the cell cycle level, since cell volume and cell cycle are correlated in most mammalian cells (Cadart et al., 2018). Cell cycle noise could in turn play a role in the dynamics of naïve pluripotency exit. For example, in human and mouse, subjecting ESCs to priming signals at different times of the cell cycle yields different outcomes in terms of lineage specification (Dalton, 2015; Gonzales et al., 2015; Liu et al., 2019) and cell cycle regulators have also been shown to directly control pluripotency (Liu et al., 2017; Michowski et al., 2020).

Finally, we show that in naïve ESCs dividing in 3D, the spindle positioning factor NuMA is expressed and localizes at spindle poles, but is not recruited to the metaphase cell cortex as is observed in HeLa cells or *Drosophila* epithelia (Kiyomitsu and Cheeseman, 2013; Kotak et al., 2012; Woodard et al., 2010). Furthermore, in cells exiting naïve pluripotency, NuMA becomes recruited to the cortex, but only in anaphase, suggesting that metaphase and anaphase localization of NuMA are regulated independently of each other. We also find that, in cells exiting naïve pluripotency, an elongated cell shape at metaphase correlates with enhanced division symmetry and preferential spindle alignment with the metaphase long axis (Figs 4 and 5). This suggests that NuMA recruitment in anaphase might be instructed by metaphase cell shape, or by the underlying mechanical forces on the cortex (Fink et al., 2011), in contrast to what has been described in cultured cells dividing in 2D and epithelia, where NuMA recruitment in metaphase is instructed by interphase cell shape (Bosveld et al., 2016; Kiyomitsu and Cheeseman, 2013). In addition to spindle positioning, NuMA regulates many other aspects of cell division, including spindle pole focusing and nucleus reformation (reviewed in Radulescu and Cleveland, 2010) making direct investigation of the effects of NuMA on spindle positioning challenging. As new tools, such as optogenetic targeting constructs, become available, it will be interesting to directly test how, for example, delocalizing NuMA from the cortex affects spindle position and division symmetry in ESC early differentiation.

Altogether, these observations suggest that spindle mobility and spindle centering by NuMA are regulated independently and are integrated by the cells to mediate spindle positioning in ESC 3D division. Our data further suggest that the mechanisms controlling division orientation and symmetry in 3D could fundamentally differ from division in 2D. More studies will be necessary to understand which of the mechanisms controlling spindle positioning in 2D environments are conserved when cells divide in 3D. Recent technical advances in the generation of 3D developmental organoids (Beccari et al., 2018; Harrison et al., 2017; Li et al., 2019; Sozen et al., 2018) will provide ideal platforms to investigate the regulation of 3D cell division.

## MATERIALS AND METHODS

### Data and materials availability

All raw data and cells used in the analysis are available upon request. This study includes no data deposited in external repositories.

### Cell lines, cell culture and drug treatments

Mouse ESCs were routinely cultured as described in Mulas et al. (2019) on 0.1% gelatin in PBS (unless otherwise stated) in N2B27 medium supplemented with 2i+LIF plus penicillin and streptomycin, at a controlled density (1.5×10^4^–3.0×10^4^ cells/cm^2^) in Falcon flasks and passaged every other day using Accutase (Sigma-Aldrich, #A6964). They were kept in 37°C incubators with 7% CO_2_. Cells were regularly tested for mycoplasma.

In this study, the cells used were embryonic day (E)14 wild-type cells and E14 cells stably expressing H2B–RFP (Cannon et al., 2015). The E14 cells stably expressing H2B–RFP were tested for contribution to chimeras, to verify that they were indeed pluripotent, by the Francis Crick Institute (London, UK) mouse facility. Cells were injected into C57BL/6 blastocysts and gave rise to a viable mouse with good contribution of the H2B–RFP cells as assessed by the color of the mouse (Fig. S2A, the mouse has extensive brown patches even though the host C57BL/6 mice are albino), and via dissection under a fluorescent lamp, which confirmed that the H2B–RFP cells contributed to tissues. All animal experiments were performed according to approved guidelines.

The culture medium was made in house, using a 1:1 DMEM/F-12 mixture (Sigma-Aldrich, #D6421-6), Neurobasal medium (Life Technologies #21103-049), 2.2 mM L-glutamine, in-house N2 (see below), B27 (Life Technologies #12587010), 3 µM Chiron (Cambridge Bioscience #CAY13122), 1 µM PD 0325901 (Sigma-Aldrich #PZ0162), LIF (Merck Millipore #ESG1107), 50 mM β-mercaptoethanol, 12.5 ng ml^−1^ insulin zinc (Sigma-Aldrich #I9278). The 200× in-house N2 stock was made using 0.791 mg ml^−1^ apotransferrin (Sigma-Aldrich #T1147), 1.688 mg ml^−1^ putrescine (Sigma-Aldrich #P5780), 3 µM sodium selenite (Sigma-Aldrich #S5261), 2.08 µg ml^−1^ progesterone (Sigma-Aldrich #P8783) and 8.8% bovine serum albumin (BSA).

Exit from naïve pluripotency was triggered by removing Chiron, PD 0325901 and LIF.

When indicated, Blebbistatin (Sigma-Aldrich #B0560) was added to the media at a final concentration of 1 µM. For Reversine treatments, Reversine (Sigma-Aldrich #R3904-1MG) was diluted in DMSO and added to the media at a final concentration of 100 nM 1 h before starting imaging. For Blebbistatin and Reversine treatments, the controls were treated with an equivalent volume of DMSO.

### Live imaging

For colony imaging, the cells were typically plated on 35 mm Ibidi dishes (IBI Scientific, #81156) coated with gelatin (unless otherwise stated) the day before the experiment, and imaged on a Perkin Elmer Ultraview Vox spinning disc (Nikon Ti attached to a Yokogawa CSU-X1 spinning disc scan head) using a C9100-13 Hamamatsu EMCCD Camera. Samples were imaged using a 60× water objective (CFI Plan Apochromat with Zeiss Immersol W oil, NA 1.2). Typically, the samples were imaged overnight acquiring a *Z*-stack with Δ*Z*=2 µm every 5 min.

Shape instability assessment (Fig. S1) and calculation of the duration of the different phases of division were undertaken by visual assessment. The shape assessment was performed blind.

### Cell size measurements

Cell volumes were measured from *Z*-stacks using the 3D mesh plugin we previously published (Smith et al., 2016; https://github.com/PaluchLabUCL/DeformingMesh3D-plugin). The far-red membrane dye CellMask™ Deep Red (Thermo Fisher Scientific, # C10046, used at 1:10,000 directly in the imaging media) was used for cell segmentation. The parameters used for segmentation were determined as optimal by visual assessment. The parameters chosen were: gamma: 1000; alpha: 5; pressure: 0; normalize: 5; image weight: 1.0×10^−4^; divisions: 3; curve weight: 0; beta: 0. The mesh deformation was made according to the perpendicular maximal gradient of the signal. The segmentation was stopped when the volume appeared resolved by visual assessment.

For 2D measurements, cell areas were measured by manually drawing the cell contour in the mid-plane of the cell using Fiji (Schindelin et al., 2012). Similarly, cell elongations were measured by drawing the cell contour in the mid-plane of the cell and automatically fitting an ellipse using Fiji and measuring the ratio of the long axis to the short axis.

### Shape instabilities assessment

To assess shape instabilities during cell division, we used four consecutive frames with a 5-min bin, the first frame being 15 min after anaphase. We first assessed in 3D which cells displayed shape instabilities over time and which displayed more stable shapes. To validate the visual analysis, we then performed a quantitative analysis of cell shape variability in 2D. We focused on the midplane of each prospective daughter cell, and used the JFilament plugin (Smith et al., 2010) to segment the cell and generate ‘snakes’ representing the outline of the cell (Fig. S3B,C). We then normalized the snakes for contour length and compared the evolution of the outlines over time, by computing a cell shape variability parameter. Specifically, we measured the curvature at each point, then the variance of this curvature over the whole snake and finally the variance of this curvature variance over the four frames assessed. Finally, we used a ROUT test (Motulsky and Brown, 2006) to identify outliers and removed them from the analysis. This led to 20 out of the 168 cells that had been visually assessed, being removed from the analysis (11 cells removed from the ‘stable’ category and nine from the ‘unstable’ category).

#### Details of curvature calculations on discretized snakes

First the snakes were scaled so that the distance was in µm and then the snakes were discretized into a succession of points separated by ∼2 µm. The curvature (*k_i_*) was then calculated at each point *i* as the rate of change of the unit tangent vector:(1)
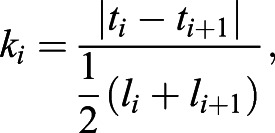
where *t*_i_ was the unit tangent from point *i−1* to point *i* and *l*_i_ the distance between points *i* and *i−1*. The curvature was negative if the cross product between the two unit tangent vectors was negative.

The average curvature was then obtained as the average over all *n* points in one frame of the snake:(2)
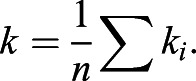
The variance of the curvature along the snake contour was then calculated as:(3)
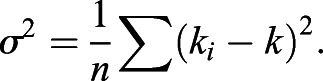
Finally, the cell shape variability parameter is then calculated as the variance of σ^2^ over time.

To measure the cell shape variability for the DMSO- and Blebbistatin-treated cells, we first excluded every cell that displayed high variability in cell area (cell area variance >100 µm^−4^), as those likely represented cells that moved extensively in the *Z*-direction. We then performed a ROUT test [using Prism (Graphpad)] to exclude outliers (two outliers for DMSO treatment, six outliers for Blebbistatin treatment).

### Transfections

Transfections were performed using 5 µg of plasmid and 6 µl of Lipofectamine, incubated in 250 µl OptiMEM for 5 min, then mixed and incubated at room temperature for 20 min, and added to cells passaged onto Ibidi dishes concomitantly. The medium was replaced with fresh medium after 5 h and the cells were imaged the next day.

For myosin-II transfection, we used a MRLC–YFP plasmid (MYL12B; kind gift from Guillaume Charras, LCN, UK). Myosin-II levels quantification was undertaken by measuring the mean gray intensity for each cell in the mid-plane of the cell at the outer, inner cortex and in the cytoplasm in interphase cells. To take into account inhomogeneities, we took three measurements per region per cell and averaged them.

### Immunofluorescence

For E-cadherin staining, cells were fixed in Ibidi dishes (IBI Scientific, #81156) in 4% formaldehyde in PHEM buffer with 0.125% Triton X-100, blocked in 3% BSA in PBS and incubated for 2 h at room temperature with primary antibodies against E-cadherin (1:200; Thermo Fisher Scientific # 13-1900) in PBS supplemented with 5% non-fat dry milk.

For NuMA staining, cells were fixed in Ibidi dishes in 10% ice cold TCA (H_2_O) at 4°C for 20 min, then permeabilized in 0.5% Triton X-100 (in PBS) for 5 mins, blocked in 3% BSA in PBS and incubated for 2 h at room temperature with primary antibodies against α-tubulin (Thermo Fisher Scientific # 62204) and NuMA (Abcam # ab36999 and # ab109262), both at 1:200, in PBS supplemented with 3% BSA.

After three PBS washes, cells were incubated for 1 h at room temperature with secondary antibodies (Alexa Fluor^®^ 647-AffiniPure donkey anti-rat IgG (Stratech Scientific, #712-605-153-JIR) and donkey anti-mouse IgG (H+L) highly cross-adsorbed secondary antibody, Alexa Fluor 488 (1:500, Thermo Fisher Scientific, #A-21202) in PBS supplemented with 3% BSA. After three PBS washes, cells were mounted using ProLong^®^ Gold Antifade Mountant with DAPI (Thermo Fisher Scientific, #P36941) or incubated for 10 min with 1:10,000 Hoechst 33342, rinsed three times and kept in PBS until imaging. Stained cells were imaged using a 63× HCX PL APO (NA 0.6–1.4) objective on a confocal microscope (Leica DMI6000 Microscope).

The levels of E-cadherin or NuMA were measured by measuring the mean gray area intensity in the mid-plane of the cell at the cell cortex and, if needed, at spindle poles. For E-cadherin, the measurement was undertaken using the freehand line tool along the cell contour either at cell–cell junction or along the outside cortex.

### Substrate coating with E-cadherin, gelatin and laminin

Ibidi dishes (IBI Scientific, #81156) were plasma activated for 30 s and incubated overnight with 50 µg ml^−1^ E-cadherin (R&D Systems, #8875-EC-050), 0.1% gelatin at room temperature or 10 µg ml^−1^ laminin (Sigma, #11243217001) at 37°C. For routine culture on gelatin, dishes were not plasma activated beforehand.

### Spindle and metaphase plate position and size measurement

The position of the metaphase plate (imaged using the H2B–RFP signal) was measured from *Z*-stacks using the ‘furrow’ option of the 3D mesh plugin (Smith et al., 2016). We confirmed that when the spindle position [using SIR-Tubulin (Tebu-bio #SC002, diluted in medium to 20 nM and incubated for 6 h)] was measured in the same cell, spindle and metaphase plate followed very similar tracks (data not shown). Briefly, the plugin allows the researcher to position a plane onto the metaphase plate and to define the center of the metaphase plate. The plugin measures the angle of the plate in the reference frame of the 3D image. The coordinates of the center of gravity of the colony is measured using the plugin by drawing a mesh around the colony using the same parameters as for volume measurements. The angle between this center of gravity and the angle of plate is then calculated.

The size of the spindle was measured by measuring the pole-to-pole distance of flat spindles.

### MSD calculation

To quantify fluctuations in metaphase plate position across conditions, we calculated the mean square displacement (MSD) as a function of time intervals, for the coordinates of the center of mass of the metaphase plate in the 3D image volume 
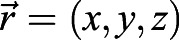
 (using the coordinate of the center of mass of the entire cell as reference, in order to quantify the relative displacement of the spindle in the reference frame of its cell). We calculated the MSD for a time interval δt as 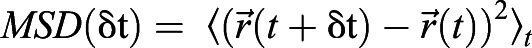, where brackets denote averaging across all time points *t* of all trajectories. We performed the same analysis for cells inside and at the periphery of colonies (*n*=19 and *n*=11 cells, respectively), as well as for cells at the periphery of 2D colonies for cells plated on E-cadherin substrates (*n*=12 cells).

### Statistical analysis

Prism 7 (Graphpad Software Inc) was used for all statistical analysis. The D'Agostino and Pearson test was used to test for the normal distribution of data. To compare means, a two-tailed one-way Student's *t*-test, a two-tailed one-way Student's *t*-test with Welch's correction or a Mann–Whitney test were performed if the data was normal with the same standard deviation, normal but with different standard deviation or not normal, respectively. One-way ANOVA or non parametric one-way ANOVA was performed to compare multiple data sets. *N*: number of independent experiments, *n*=number of points (not stated for dot plots).

## Supplementary Material

Supplementary information

Reviewer comments
