# Supplementary material for: Three-dimensional geometry controls division symmetry in stem cell colonies
Source: J Cell Sci. 2021 Jul 29;134(14):jcs255018. doi: 10.1242/jcs.255018 (PMC8349555; doi:10.1242/jcs.255018)
Supplement: Supplementary information [file joces-134-255018-s1.pdf]

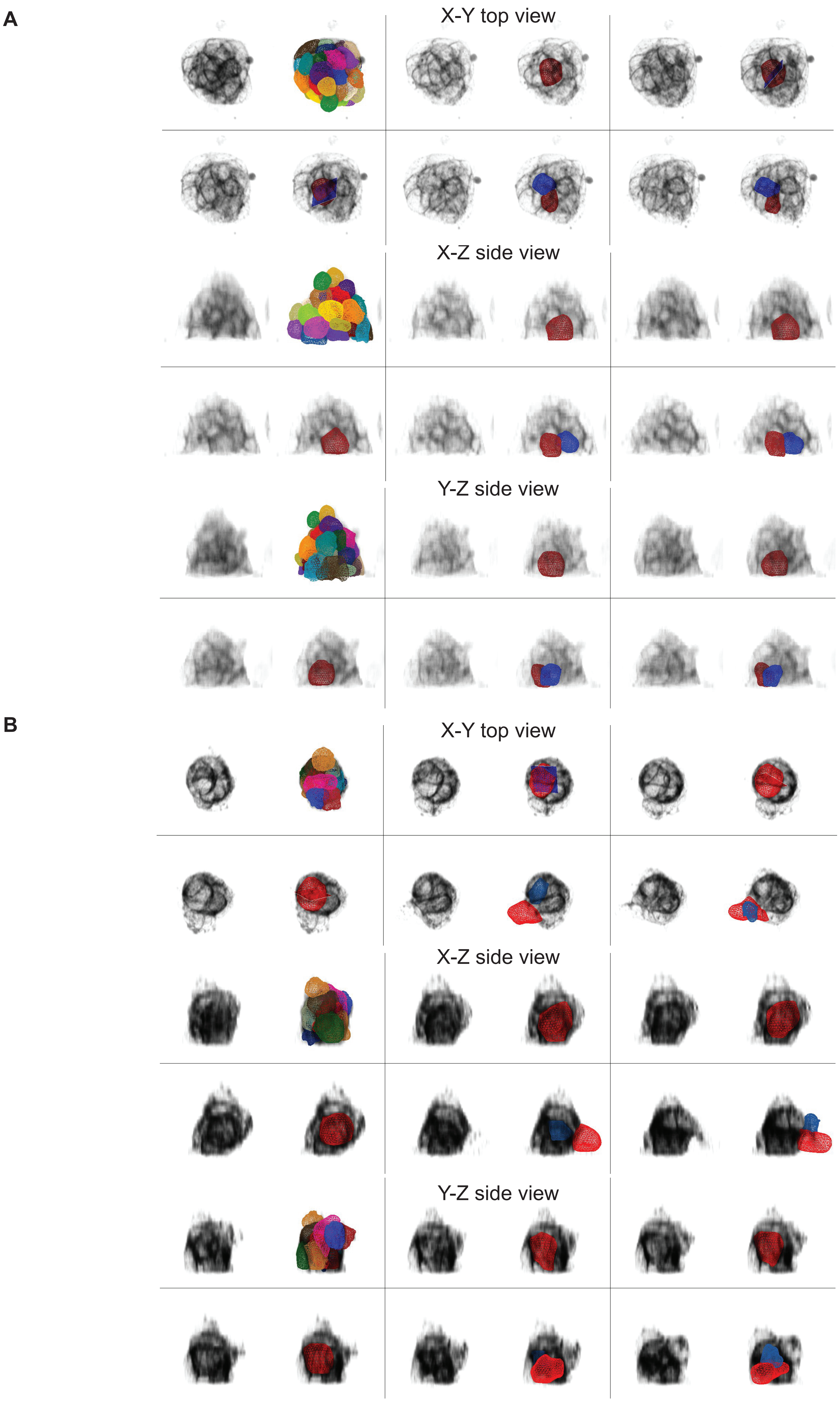

Journal of Cell Science • Supplementary information

Fig. S1. ESC divisions in 3D colonies.

A) 3D rendition of an ESC colony with one cell dividing inside the colony over time. For each time point, the left frame shows a 3D rendition of the membrane staining (CellMask^{TM}, black) and the right frame shows a 3D segmentation of the dividing cell (except for the first timepoint, where a 3D segmentation of all the cells in the colony is shown). One picture is shown every 10 min. The top panel shows a top view; at the 3rd and 4th timepoints, a blue plane highlights the position and orientation of the metaphase plate, identified from the H2B-RFP signal (not displayed). The middle and bottom panels show the two orthogonal side views.

B) 3D rendition of an ES colony with one cell dividing at the periphery of the colony over time. For each time point, the left frame shows a 3D rendition of the membrane staining (CellMask^{TM}, black) and the right frame shows a 3D segmentation of the dividing cell (except for the first timepoint, where a 3D segmentation of all the cells in the colony is shown). One picture is shown every 10 min. The top panel shows a top view; at the 3rd and 4th timepoints, a blue plane highlights the position and orientation of the metaphase plate, identified from the H2B-RFP signal (not displayed). The middle and bottom panels show the two orthogonal side views.

Journal of Cell Science • Supplementary information

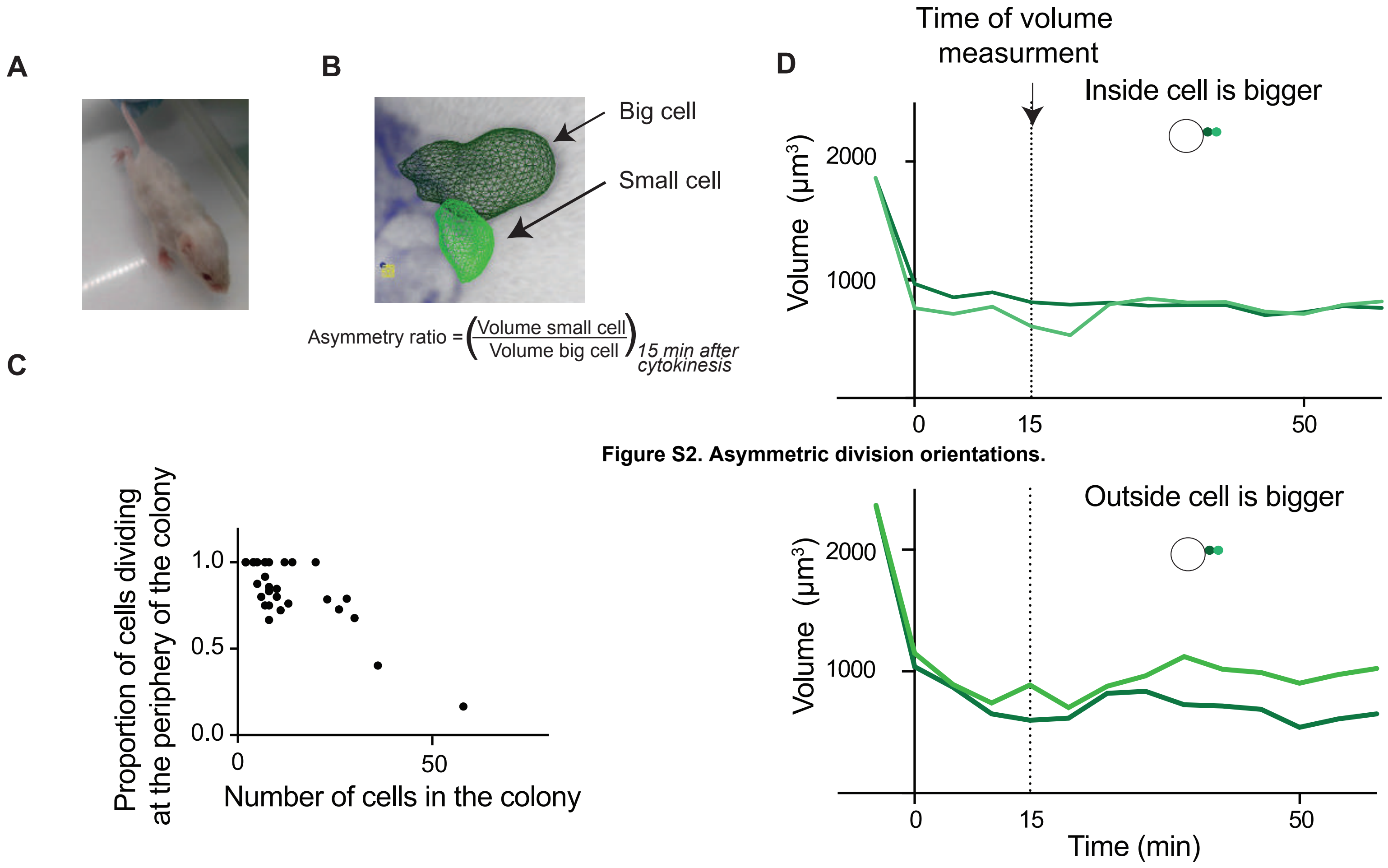

Fig. S2. Asymmetric division orientations.

A) Picture of a chimera mouse obtained by injection of the H2B-RFP ESCs into the blastocyst of an albino C57BL/6 mouse; the mouse displays brown patches even though the host blastocyst came from an albino mouse, indicating good integration of the injected cells.

B) Image: 3D rendition of 2 daughter cells labeled with CellMask™ deep red and segmented using the 3D mesh plugin (see Methods). Bottom: definition of the asymmetry ratio.

C) Dot plot showing the proportion of cells dividing at the periphery of the colony as a function of the number of cells in the colony. N=5.

D) Example plots showing the evolution of the volumes of daughter cells after division at the periphery of the colony, in cases where the inside cell is the bigger one (left) or the smaller one (right). 0 is the time of cytokinesis. The time when the asymmetry ratio reported in Figure 1D is measured (15 min) is highlighted with a dashed line.

Journal of Cell Science • Supplementary information

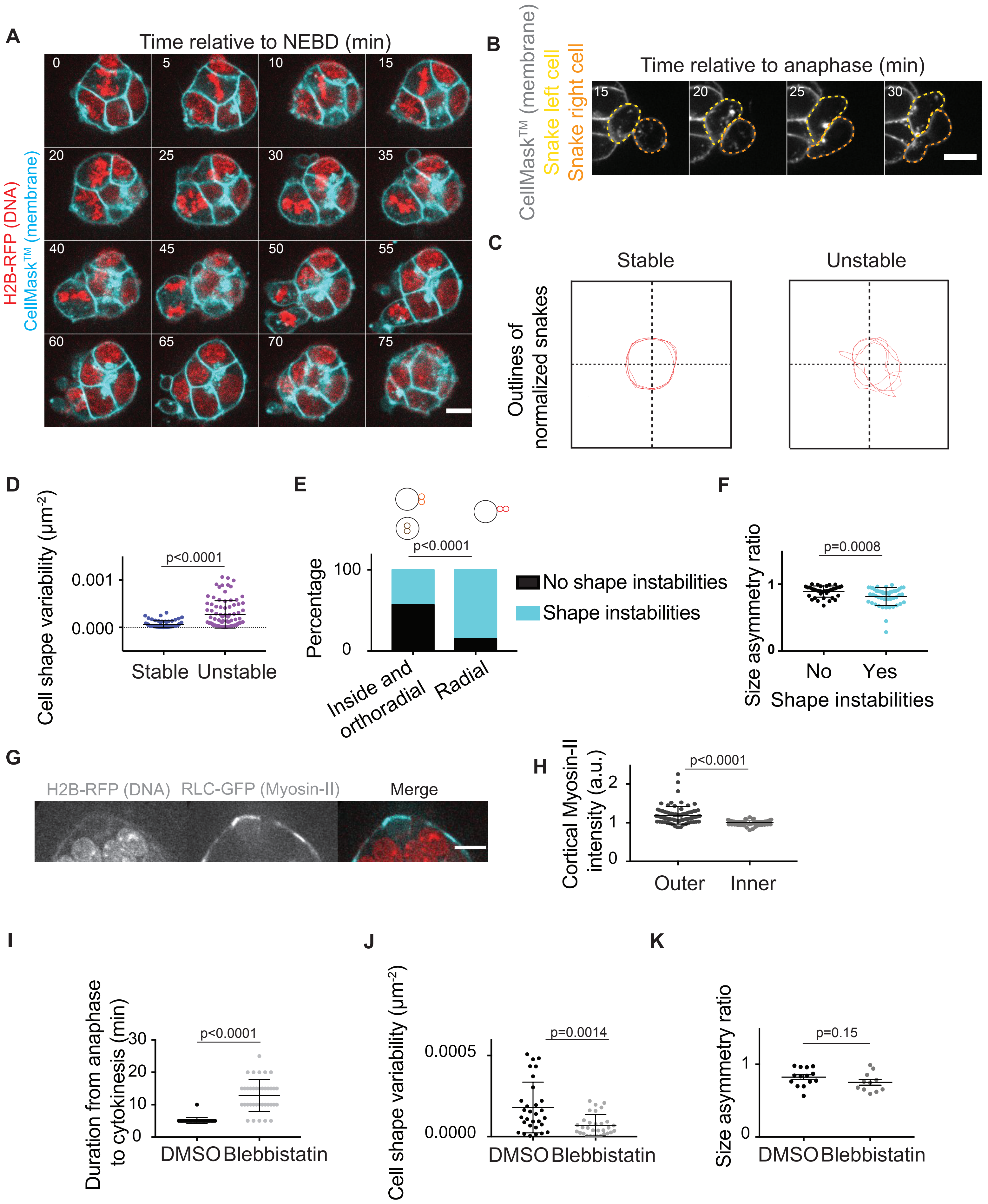
A
Time relative to NEBD (min)
H2B-RFP (DNA)
CellMask™ (membrane)
0
5
10
15
20
25
30
35
40
45
50
55
60
65
70
75
B
CellMask™ (membrane)
Snake left cell
Snake right cell
Time relative to anaphase (min)
15
20
25
30
C
Outlines of normalized snakes
Stable
Unstable
D
Cell shape variability (μm⁻²)
0.001
0.000
p<0.0001
Stable
Unstable
E
Percentage
100
0
p<0.0001
No shape instabilities
Shape instabilities
Inside and orthoradial
Radial
F
Size asymmetry ratio
p=0.0008
1
0
No
Yes
Shape instabilities
G
H2B-RFP (DNA)
RLC-GFP (Myosin-II)
Merge
H
Cortical Myosin-II intensity (a.u.)
p<0.0001
2
1
0
Outer
Inner
I
Duration from anaphase to cytokinesis (min)
p<0.0001
30
20
10
0
DMSO
Blebbistatin
J
Cell shape variability (μm⁻²)
p=0.0014
0.0005
0.0000
DMSO
Blebbistatin
K
Size asymmetry ratio
p=0.15
1
0
DMSO
Blebbistatin

Fig. S3. Polar contractions do not cause size asymmetries at cell division.

A) Representative confocal time-lapse of an ESC 3D colony expressing H2B-RFP (red) and labeled with CellMask™ deep red (cyan) showing shape instabilities during cell division at the periphery of the colony. Time in minutes; 0 is NEBD. One Z plane is shown. Scale bar: 10 μm.

B) Representative confocal time-lapse of two ESCs expressing H2B-RFP and labeled with CellMask™ deep red (cyan) showing the membrane outline as used to create the 2 snakes (yellow and orange) with the JFilament plugin for 2D segmentation of the prospective daughter cells. Time in minutes; 0 is anaphase. One Z plane is shown. Scale bar: 10 μm.

C) Representative examples of snakes used to analyze cell shape stability. The two examples correspond to cell shapes classified as “stable” (left) and “unstable” (right) through visual assessment of 3D stacks. Snakes are normalized for length and the graphs are centered at the center of the snakes.

D) Dot plot showing the cell shape variability (defined as the variance of the curvature variance, see Materials and Methods) for snakes corresponding to prospective daughter cells visually classified as “stable” (blue) or “unstable” (purple). The mean and standard deviations are shown. N=3.

E) Bar graph showing the percentage of cells displaying shape instabilities (blue) or no shape instabilities (black) as assessed visually and confirmed using the automated snake-based analysis (panels B-D), for H2B-RFP ESCs dividing inside or at the periphery of a colony with the spindle parallel to the colony border (inside and orthoradial, left), or at the periphery of a colony with the spindle perpendicular to the colony border (radial, right). N=3, n= 84.

F) Dot plot showing the asymmetry ratio between daughter cells for H2B-RFP expressing ESCs displaying (right) or not displaying (left) shape instabilities. The mean and standard deviation are shown. N=3.

G) Representative confocal images of the border of a colony of ESCs expressing H2BRFP and RLC-GFP. Scale bar: 10 μm.

H) Dot plot showing cortical Myosin-II intensity in ESCs expressing RLC-GFP. The mean intensity (normalized to the cytoplasmic intensity) at the outer cortex (left) and inner cortex (at cell-cell junctions, right), and standard deviation are shown. N=2.

I) Dot plot showing the time between anaphase and cytokinesis in ESCs treated with DMSO (black) and with 1 μm. Blebbistatin (gray). The mean and standard deviations are shown. N=2.

J) Dot plot showing the cell shape variability (defined as the variance of the curvature variance, see Methods) of snakes for ESCs dividing at the periphery of a colony treated with DMSO (left) or 1 ìM Blebbistatin (right). The mean and standard deviations are shown. N=2 colony treated with DMSO and 1 ìM Blebbistatin. The mean and standard deviation are shown. N=2.

K) Dot plot showing the asymmetry ratio for ESCs dividing at the periphery of the colony treated with DMSO and 1 μM Blebbistatin. The mean and standard deviation are shown. N=2.

Journal of Cell Science • Supplementary information

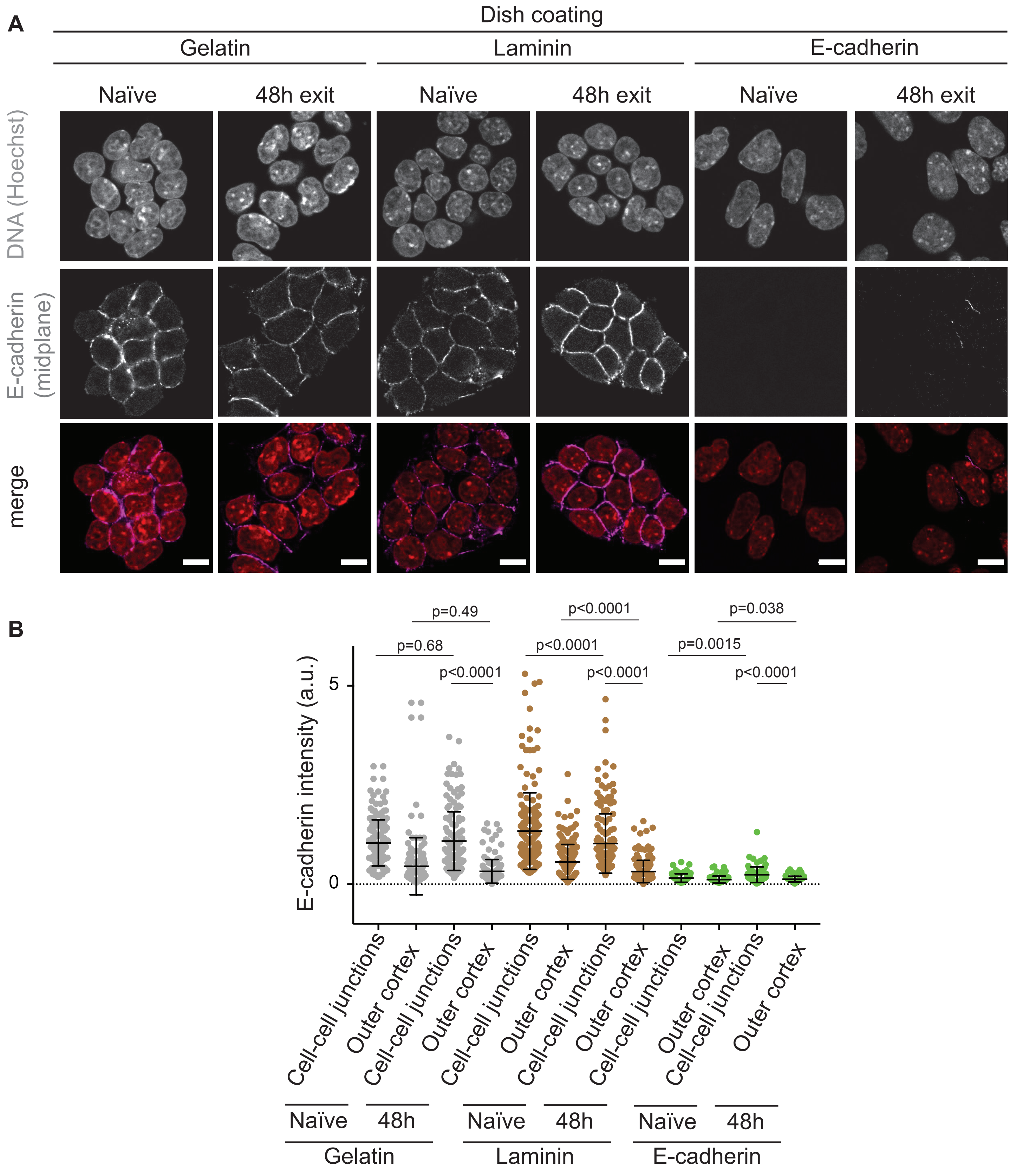

Fig. S4. E-cadherin distribution in cells plated on different substrates.

A) Representative confocal images of H2B-RFP expressing naïve ESCs (left), and cells allowed to exit naïve pluripotency for 48h (right), plated on gelatin (left panel), laminin (middle panel) and E-cadherin (right panel) and stained for DNA and E-cadherin. The midplane is shown. Scale bars: 10 µm.

B) Dot plot showing the intensity levels of E-cadherin staining in H2B-RFP expressing naïve ESCs (left) or cells allowed to exit naïve pluripotency for 48h (right), at cell-cell junctions and at cell borders exposed to the outside of the colony (outer cortex) for cells plated on gelatin (gray dots), laminin (brown dots) and E-cadherin (green dots). The mean and standard deviation are shown. N=3.

Journal of Cell Science • Supplementary information

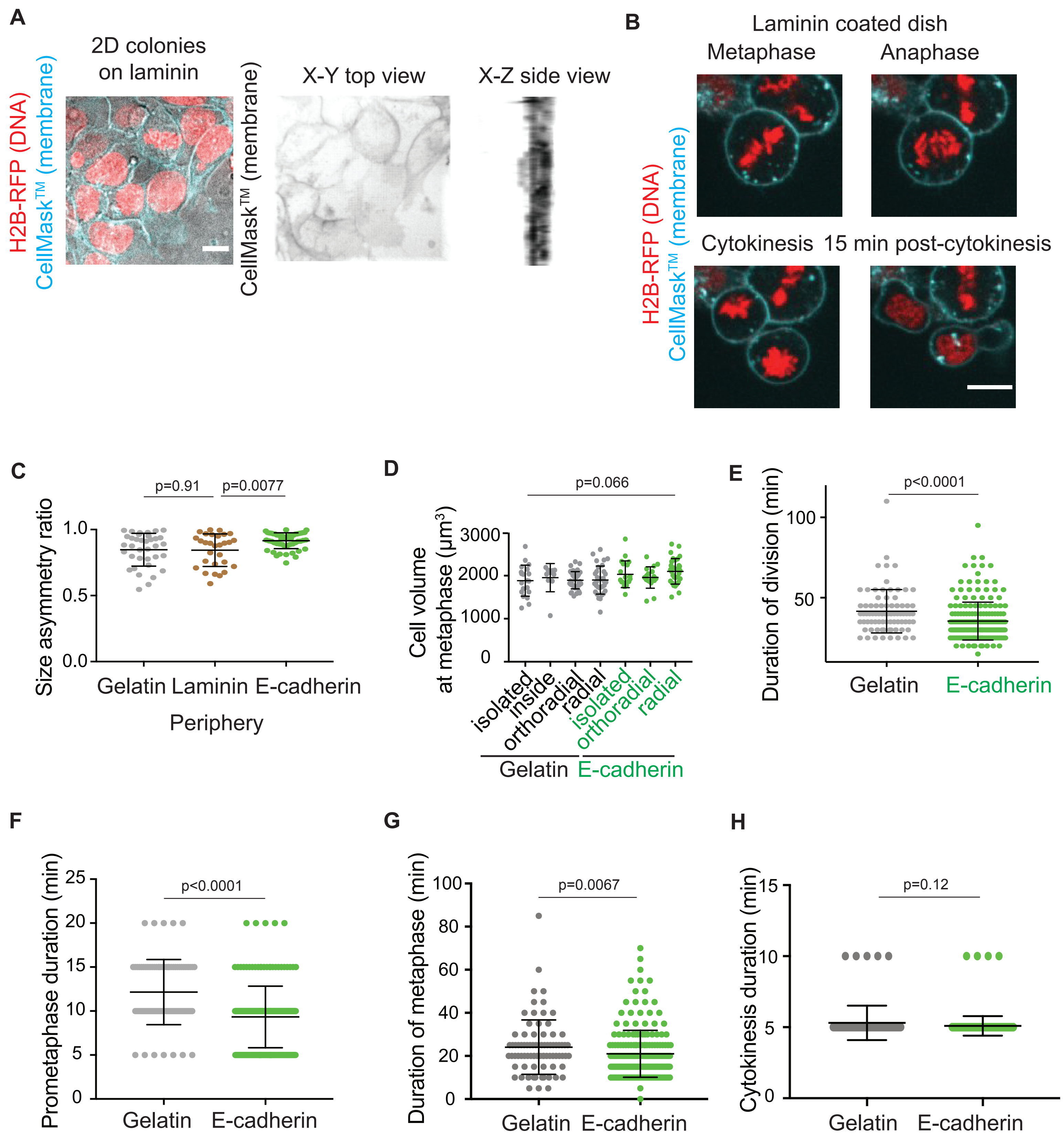
A
2D colonies on laminin
H2B-RFP (DNA)
CellMask™ (membrane)
X-Y top view
X-Z side view
CellMask™ (membrane)
B
Laminin coated dish
Metaphase
Anaphase
Cytokinesis
15 min post-cytokinesis
H2B-RFP (DNA)
CellMask™ (membrane)
C
p=0.91
p=0.0077
Size asymmetry ratio
1.0
0.5
0.0
Gelatin
Laminin
E-cadherin
Periphery
D
p=0.066
Cell volume at metaphase (µm³)
3000
2000
1000
0
isolated
inside
orthoradial
radial
isolated
orthoradial
radial
Gelatin
E-cadherin
E
p<0.0001
Duration of division (min)
100
50
Gelatin
E-cadherin
F
p<0.0001
Prometaphase duration (min)
25
20
15
10
5
0
Gelatin
E-cadherin
G
p=0.0067
Duration of metaphase (min)
100
80
60
40
20
0
Gelatin
E-cadherin
H
p=0.12
Cytokinesis duration (min)
15
10
5
0
Gelatin
E-cadherin

Figure S5. Cell spreading is not sufficient to increase division symmetry in ESCs.

A) Representative images of colonies of ESCs expressing H2B-RFP (red) and labeled with CellMask™ deep red (cyan) on a laminin-coated substrate. A Z-projection overlaid on a transmitted light image is shown on the left and 3D renditions of a top view (XY, middle) and a side view (YZ, right) are shown on the right. Scale bar: 10 µm.

B) Representative time-lapse of a dividing ESC expressing H2B-RFP (red) and labeled with CellMask™ deep red (cyan) on a laminin-coated substrate. One Z-plane is shown. Scale bar: 10 µm.

C) Dot plot showing the asymmetry ratio for ESCs dividing on gelatin (control), laminin or E-cadherin (E-cadherin data are pooled datapoints from Figure 3E, replotted here as reference). The mean and standard deviation are shown. N=3.

D) Dot plot showing the volumes (measured from 3D stacks) of ESCs in metaphase for cells in 3D colonies on gelatin (gray) or plated on E-cadherin (green). The mean and standard deviation are shown. N=2.

E) Dot plot showing the total duration of the division from NEBD to cytokinesis for cells in 3D colonies on gelatin (gray) or plated on E-cadherin (green). The mean and standard deviation are shown. N=2.

F) Dot plot showing the duration of prometaphase for cells in 3D colonies on gelatin (gray) or plated on E-cadherin (green). The mean and standard deviation are shown. N=2.

G) Dot plot showing the duration of metaphase for cells in 3D colonies on gelatin (gray) or plated on E-cadherin (green). The mean and standard deviation are shown. N=2.

H) Dot plot showing the duration of cytokinesis for cells in 3D colonies on gelatin (gray) or plated on E-cadherin (green). The mean and standard deviation are shown. N=2.

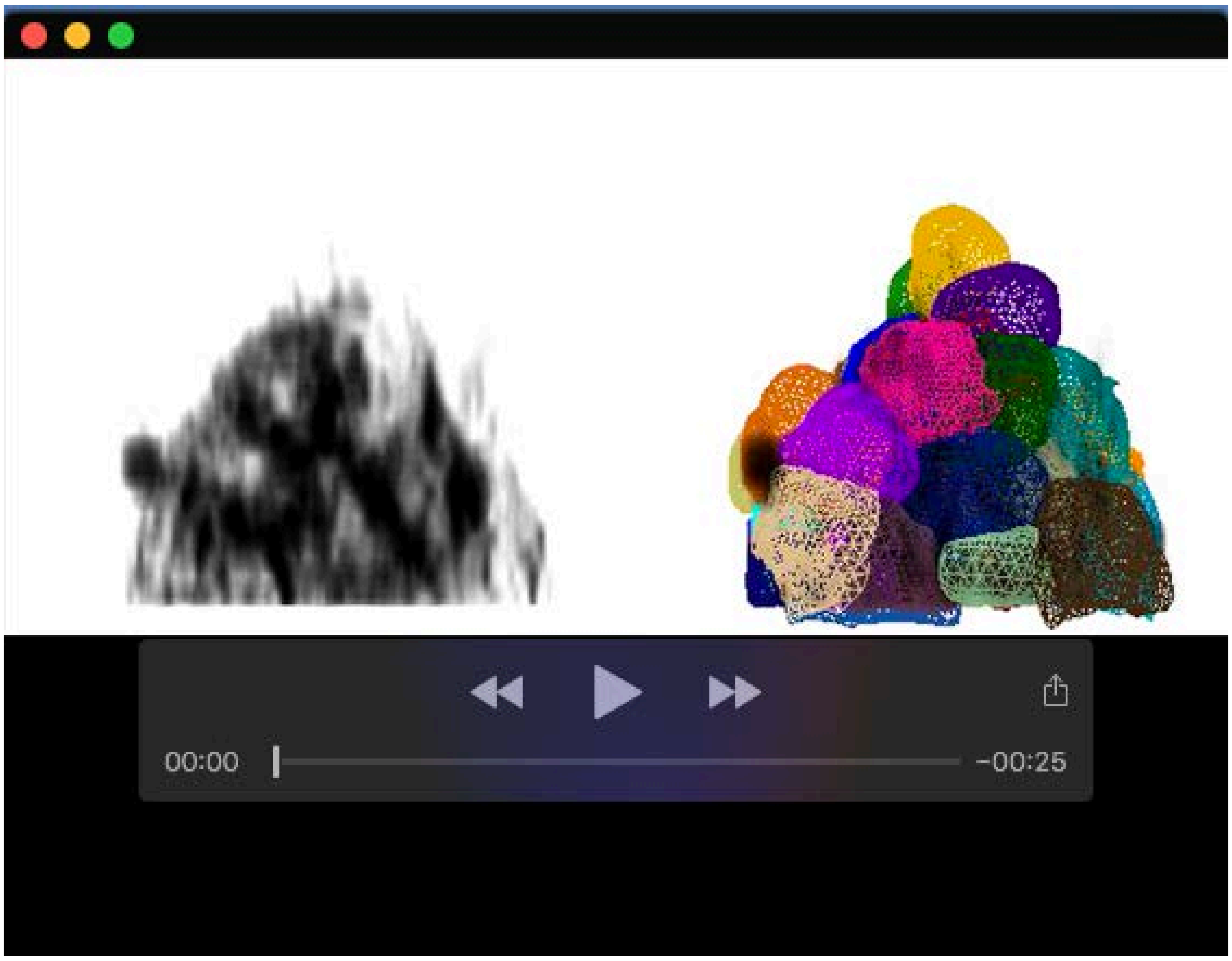

Movie 1. 3D rendition of an ESC colony with one cell dividing inside the colony.

The left frame shows a 3D rendition of the membrane staining (CellMask^{TM}, black) and the right frame shows a 3D segmentation of the colony.

Journal of Cell Science • Supplementary information

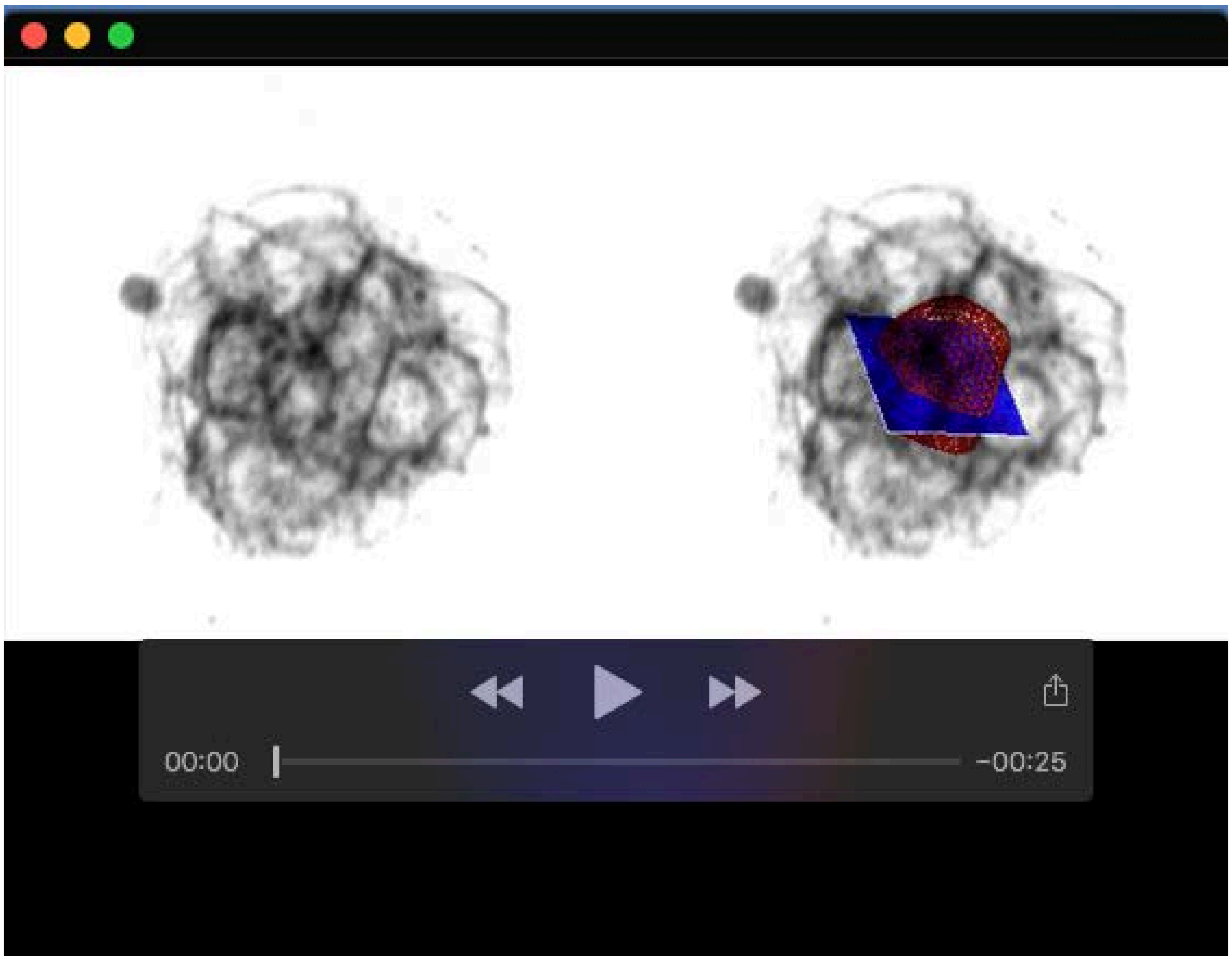

Movie 2. Time-lapse of a 3D rendition of an ESC colony with one cell dividing inside the colony over time.

For each time point, the left frame shows a 3D rendition of the membrane staining (CellMask^{TM}, black) and the right frame shows a 3D segmentation of the dividing cell. One picture is shown every 5 min. The first frame shows a 3D segmentation of all the cells in the colony. The blue plane highlights the position and orientation of the metaphase plate, identified from the H2B-RFP signal (not displayed).

Journal of Cell Science • Supplementary information

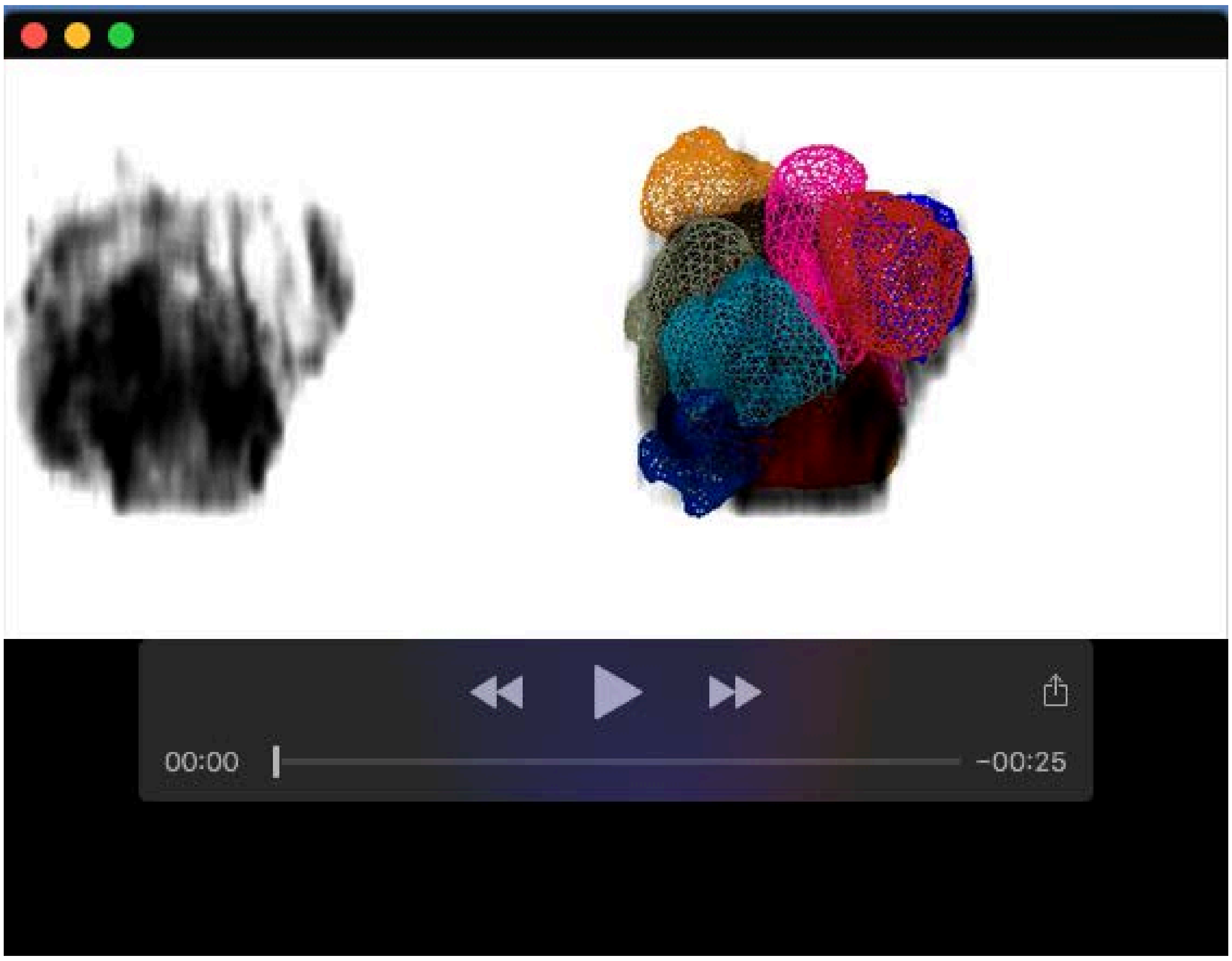

Movie 3. 3D rendition of an ESC colony with one cell dividing at the periphery of the colony.

The left frame shows a 3D rendition of the membrane staining (CellMask^{TM}, black) and the right frame shows a 3D segmentation of the colony.

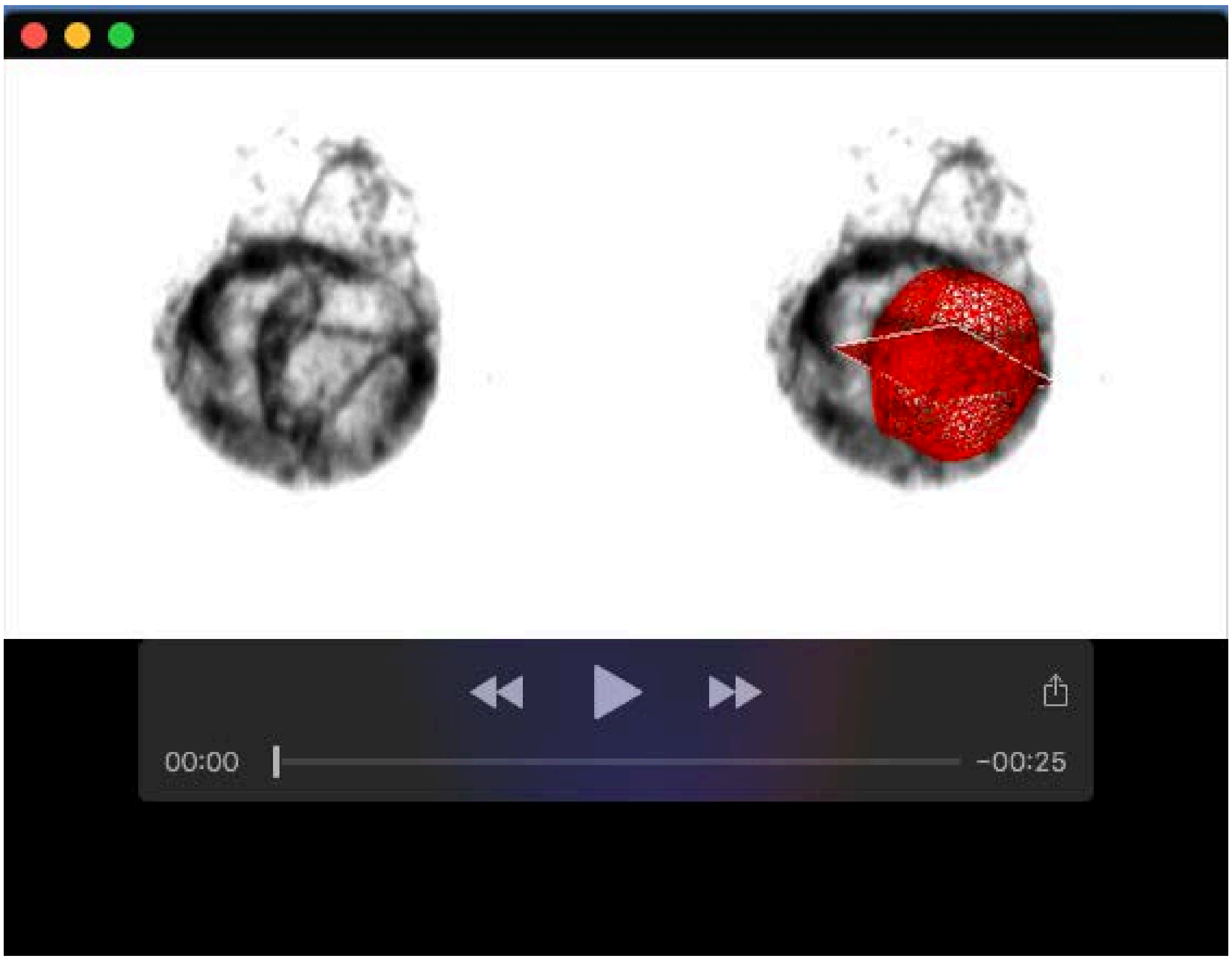

Movie 4. Time-lapse of a 3D rendition of an ESC colony with one cell dividing at the periphery of the colony over time.

For each time point, the left frame shows a 3D rendition of the membrane staining (CellMask^{TM}, black) and the right frame shows a 3D segmentation of the dividing cell. One picture is shown every 5 min. The first frame shows a 3D segmentation of all the cells in the colony. The blue plane highlights the position and orientation of the metaphase plate, identified from the H2B-RFP signal (not displayed).

Journal of Cell Science • Supplementary information

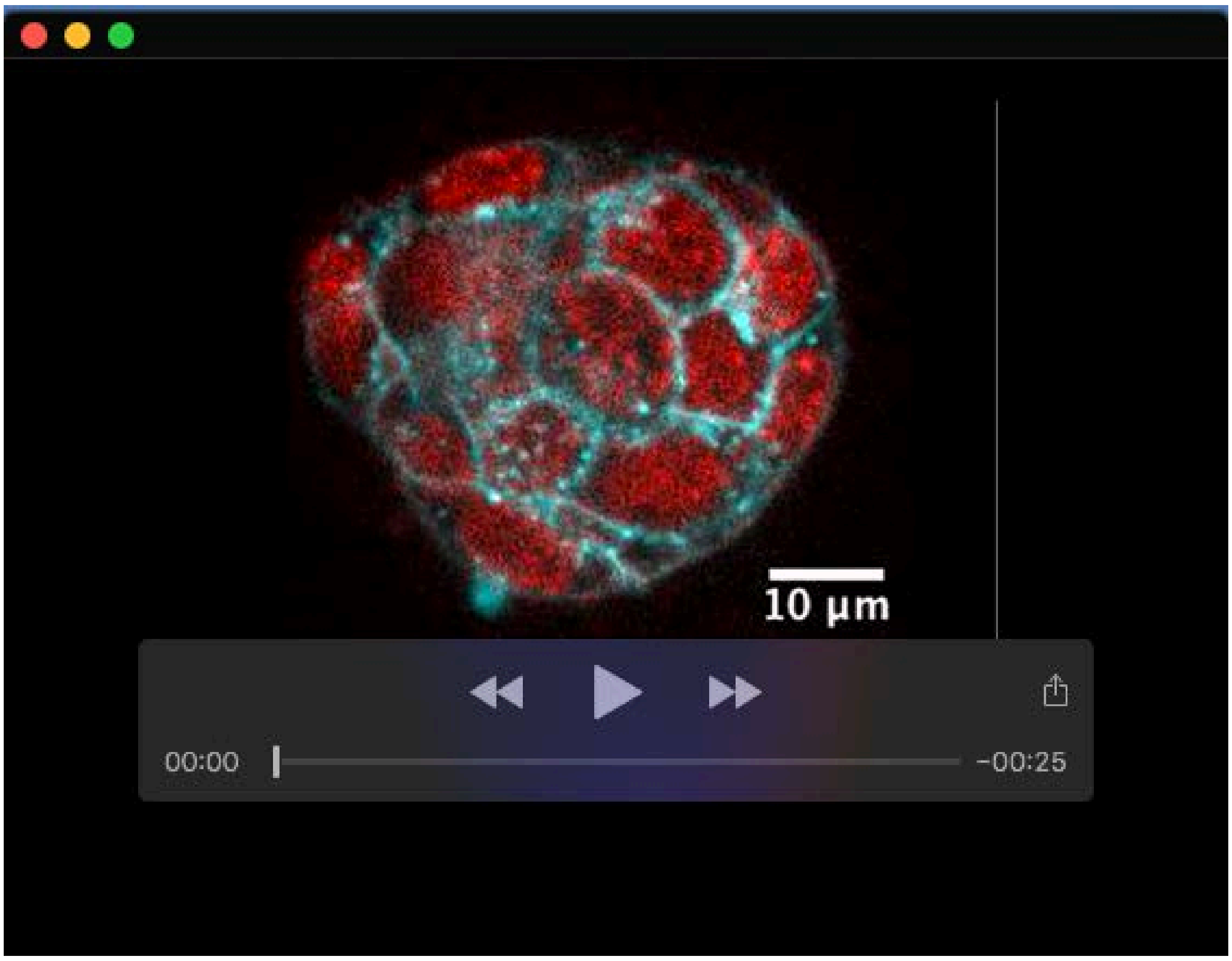

Movie 5. ESC dividing inside a colony.

Time-lapse (spinning-disk confocal microscopy) of a H2B-RFP (red) expressing naïve ESC colony labeled with CellMask^{TM} deep red (cyan) with one cell dividing symmetrically inside the colony. One frame is shown every 5 min. One Z plane is shown. Scale bar: 10 µm.

Journal of Cell Science • Supplementary information

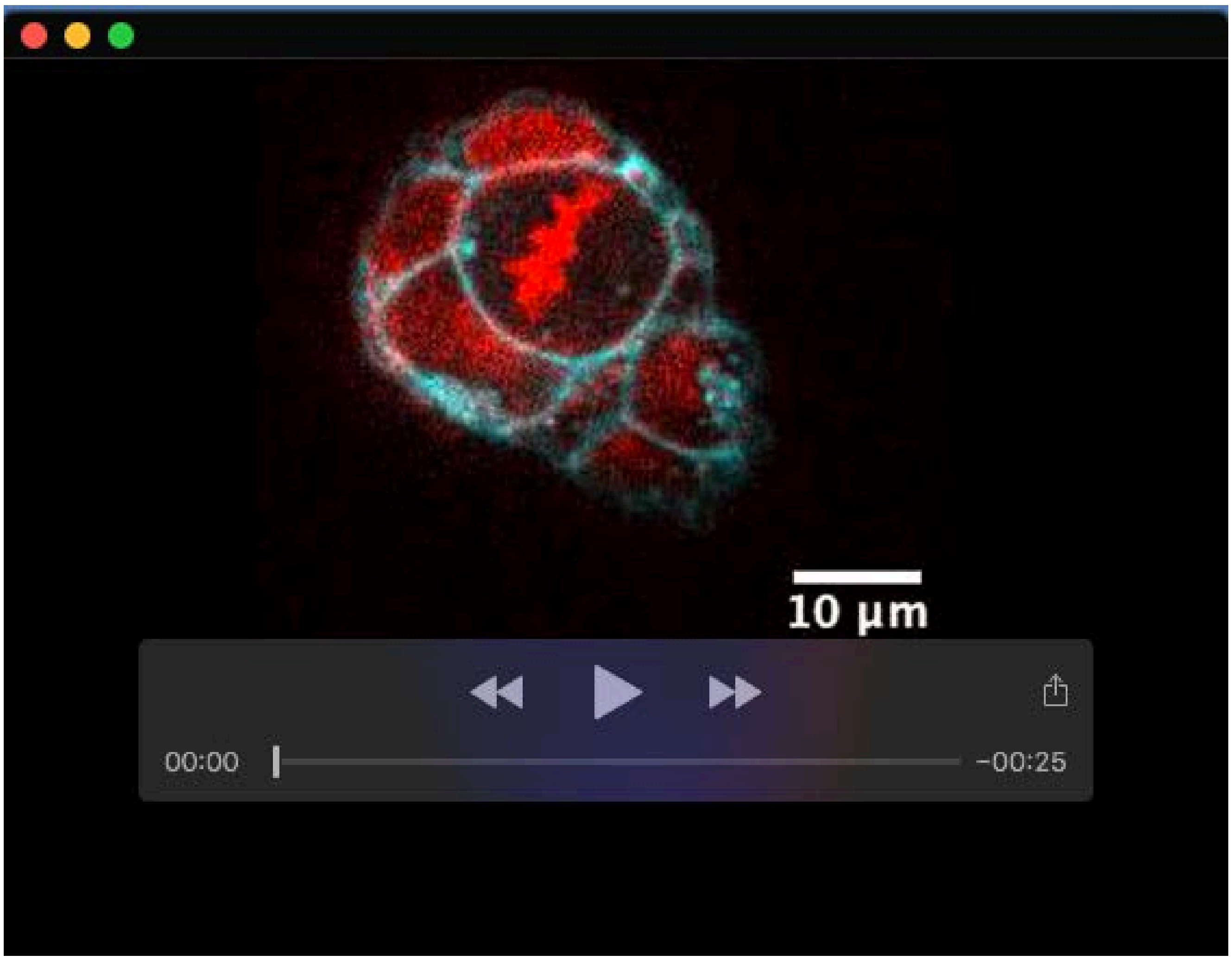

Movie 6. ESC dividing at the periphery of a colony.

Time-lapse (spinning-disk confocal microscopy) of a H2B-RFP (red) expressing naïve ESC colony labeled with CellMask^{TM} deep red (cyan) with one cell dividing asymmetrically at the periphery of the colony. One frame is shown every 5 min. One Z plane is shown. Scale bar: 10 µm.

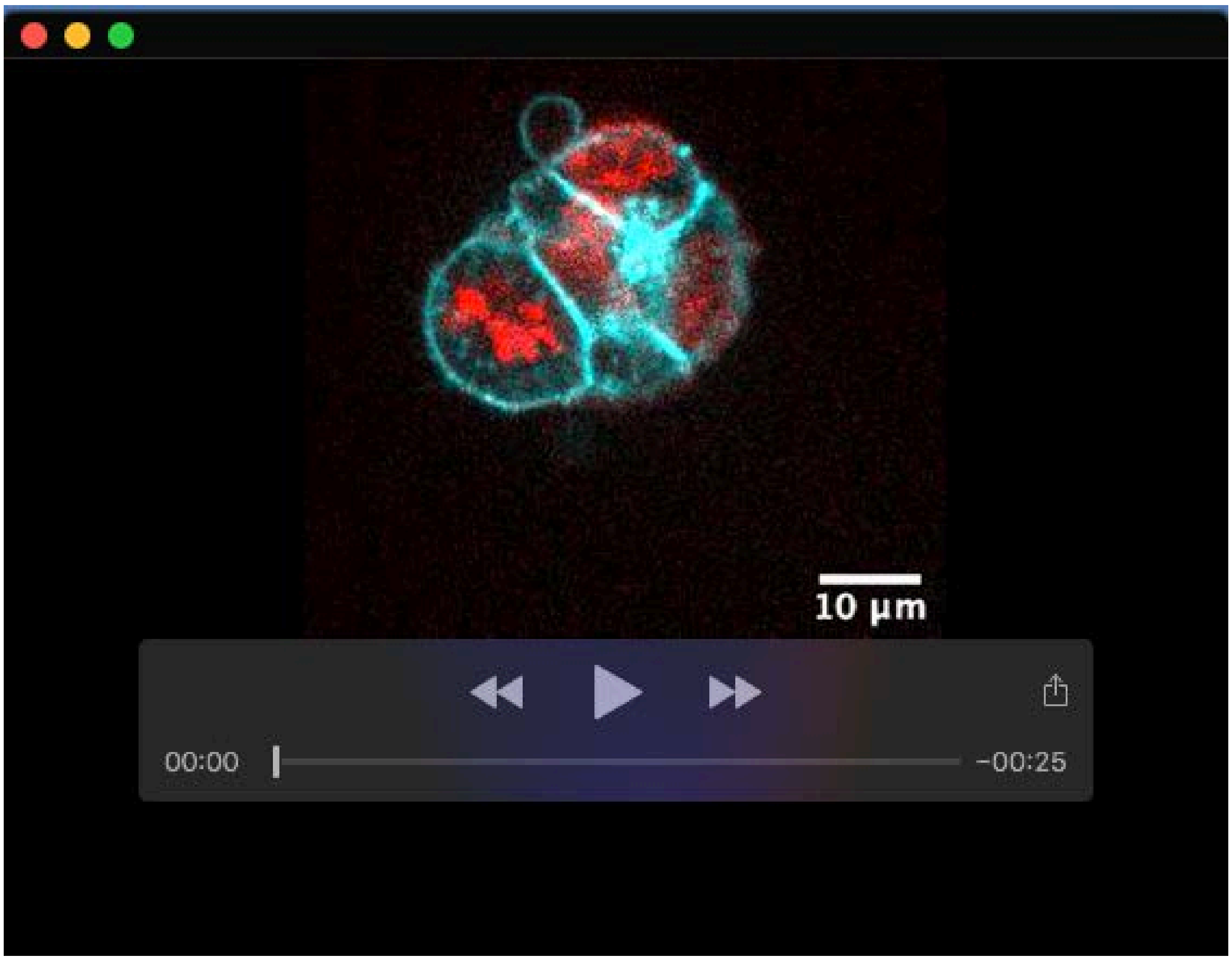

Movie 7. Shape instabilities during ESC divisions at the periphery of a colony.

Time-lapse (spinning-disk confocal microscopy) of a colony of ESCs expressing H2B-RFP (red) and labeled with CellMask™ deep red (cyan) showing shape instabilities in cells dividing at the periphery of the colony. One frame is shown every 5 min. One Z plane is shown. Scale bar: 10 µm.

Journal of Cell Science • Supplementary information

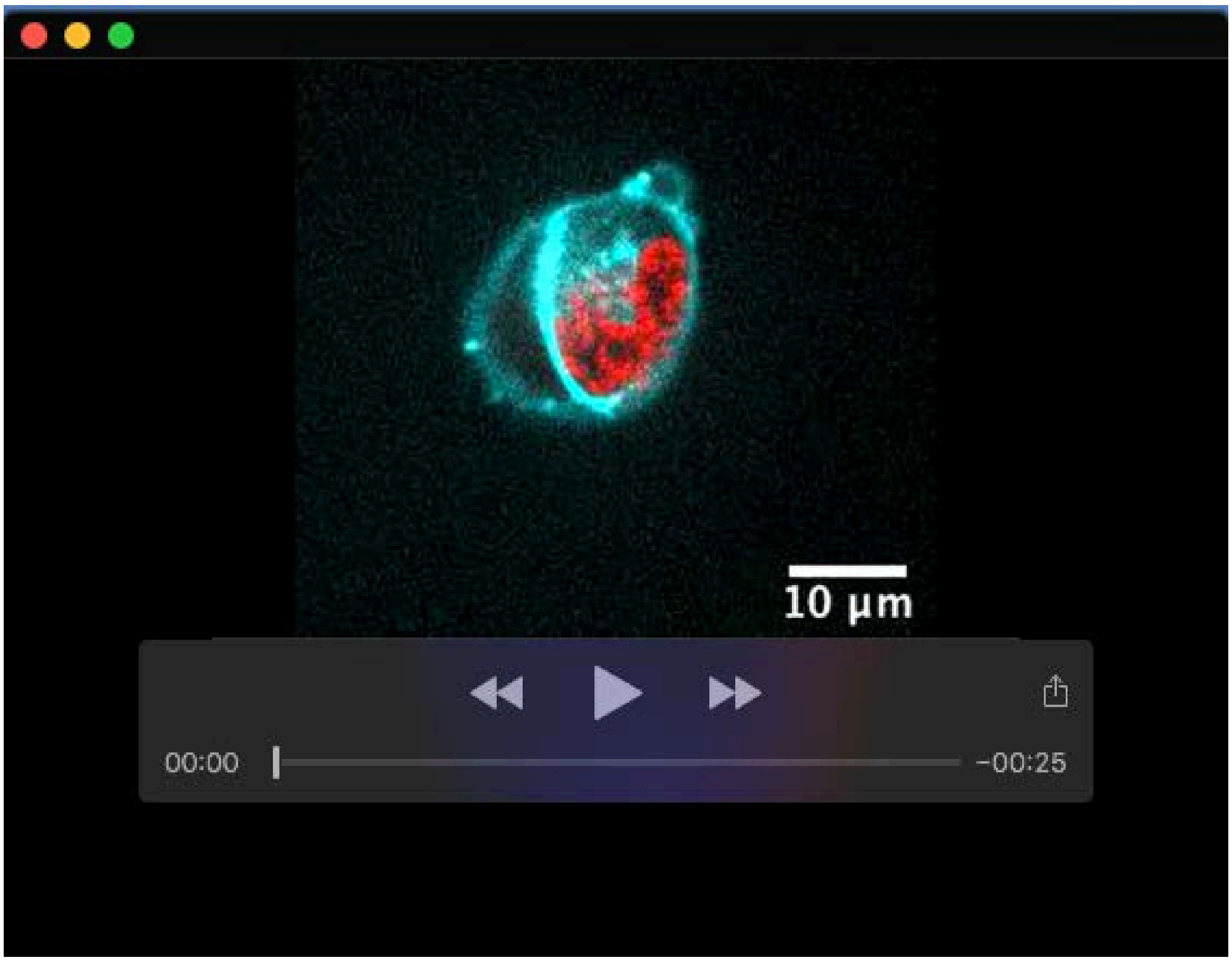

Movie 8. Example of blebbistatin-treated ESC dividing at the periphery of a colony. Time-lapse (spinning-disk confocal microscopy) of a colony of ESCs expressing H2B-RFP (red) and labelled with CellMask^{TM} deep red (cyan) and treated with 1 µM Blebbistatin with one cell dividing at the periphery of the colony. The cell displays strong size asymmetry between daughter cells. One frame is shown every 5 min. One Z plane is shown. Scale bar: 10 µm.

Journal of Cell Science • Supplementary information

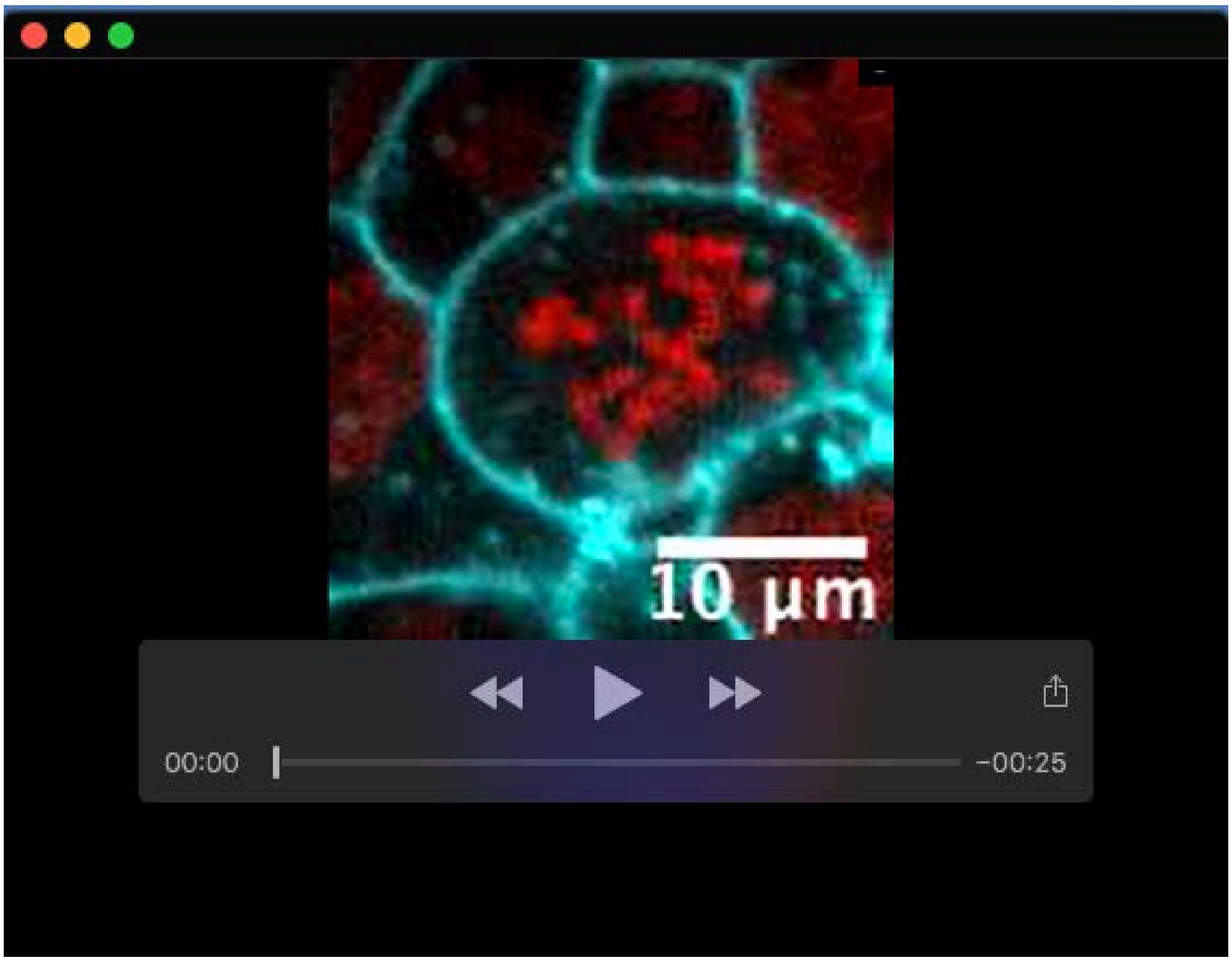

Movie 9. Stable spindle in an ESC dividing inside a colony.

Time-lapse (spinning-disk confocal microscopy) of a H2B-RFP (red) expressing ESC colony labeled with CellMask^{TM} deep red (cyan) with one cell dividing inside the colony. One frame is shown every 2 min. One Z plane is shown. Scale bar: 10 µm.

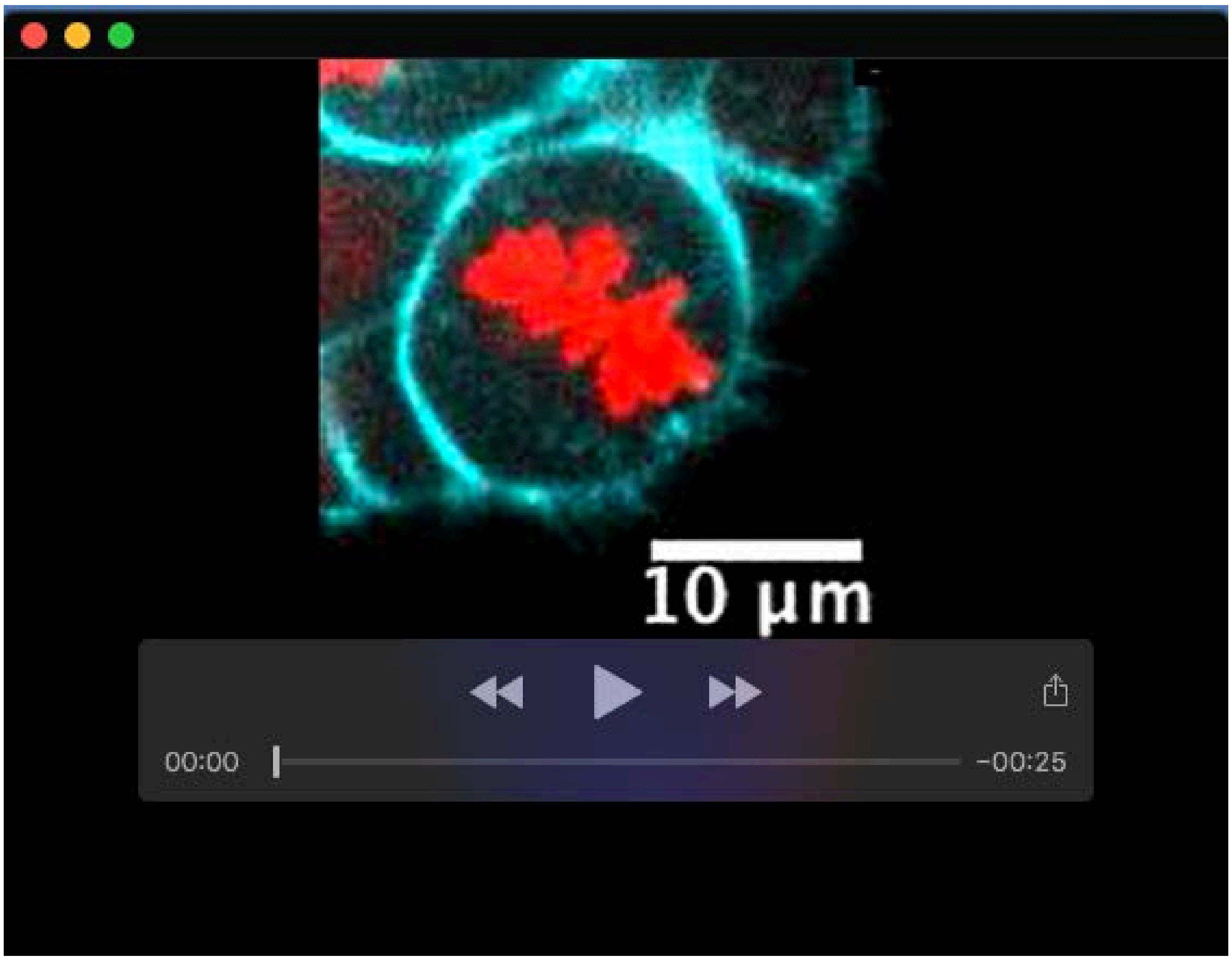

Movie 10. Unstable spindle in an ESC dividing at the periphery of a colony.

Time-lapse (spinning-disk confocal microscopy) of a H2B-RFP (red) expressing ESC colony labeled with CellMask^{TM} deep red (cyan) with one cell dividing at the periphery of the colony. One frame is shown every 2 min. One Z plane is shown. Scale bar: 10 μm.

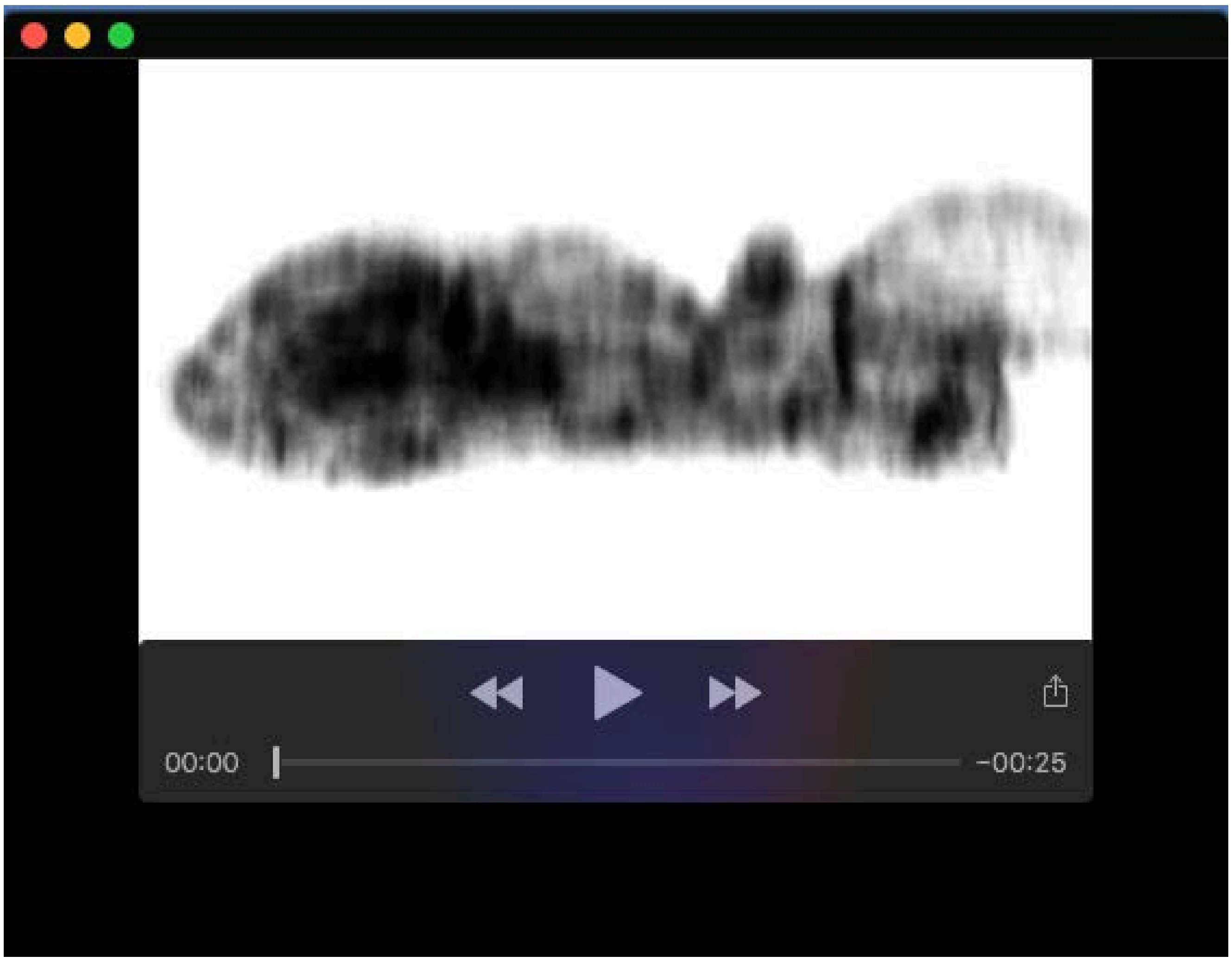

Movie 11. 3D rendition of an ESC colony plated on E-cadherin.

360˚ rotation of a 3D rendition of the membrane staining (CellMask™, black) of a colony of ESCs plated on E-cadherin.

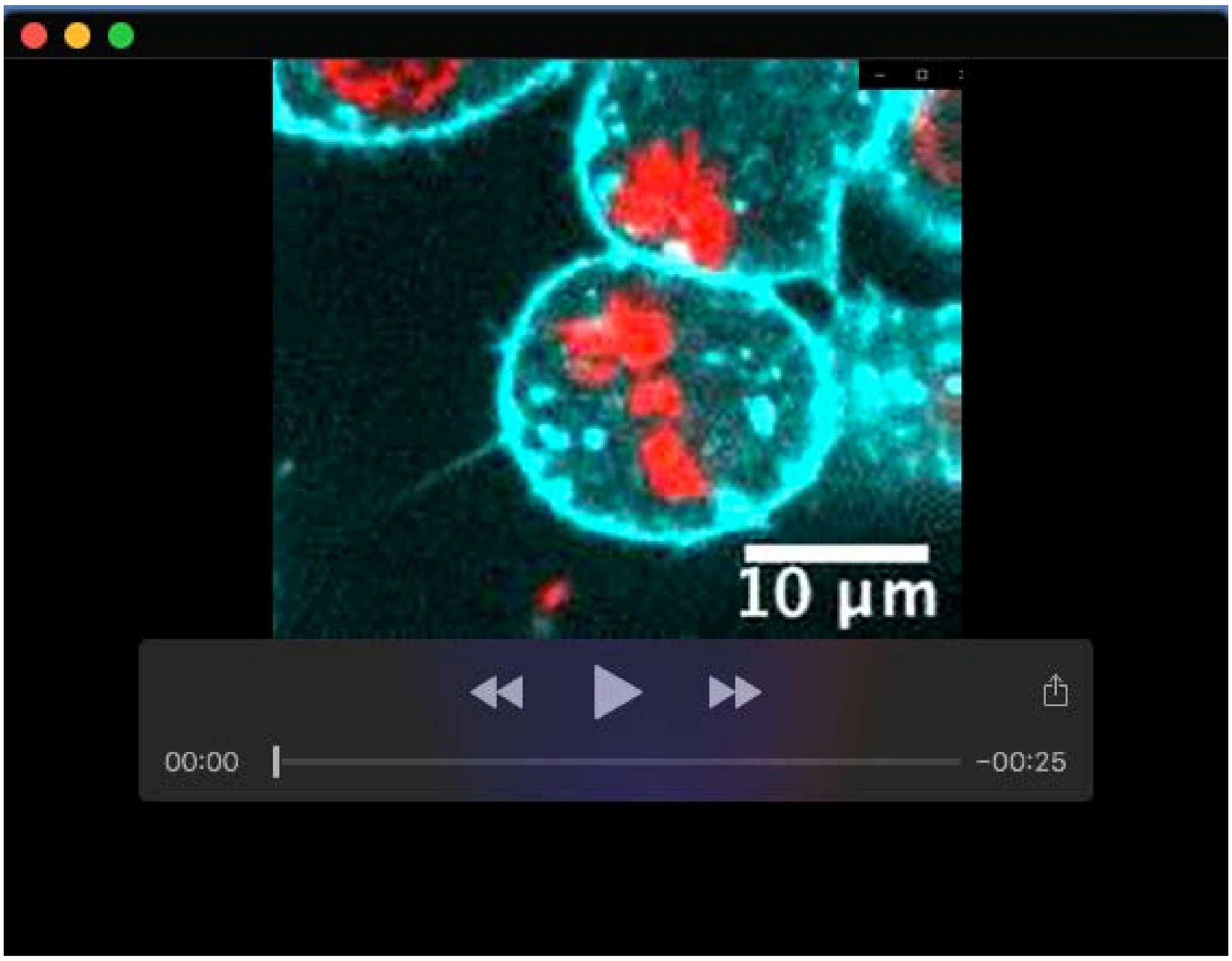

Movie 12. Example of an ESC dividing at the periphery of a 2D colony plated on E-cadherin.

Time-lapse (spinning-disk confocal microscopy) of a colony of ESCs expressing H2B-RFP (red) plated on E-cadherin and labeled with CellMask^{TM} deep red (cyan), with one cell dividing at the periphery of the colony. Cell division appears symmetric. One frame is shown every 5 min. One Z plane is shown. Scale bar: 10 µm.

Journal of Cell Science • Supplementary information

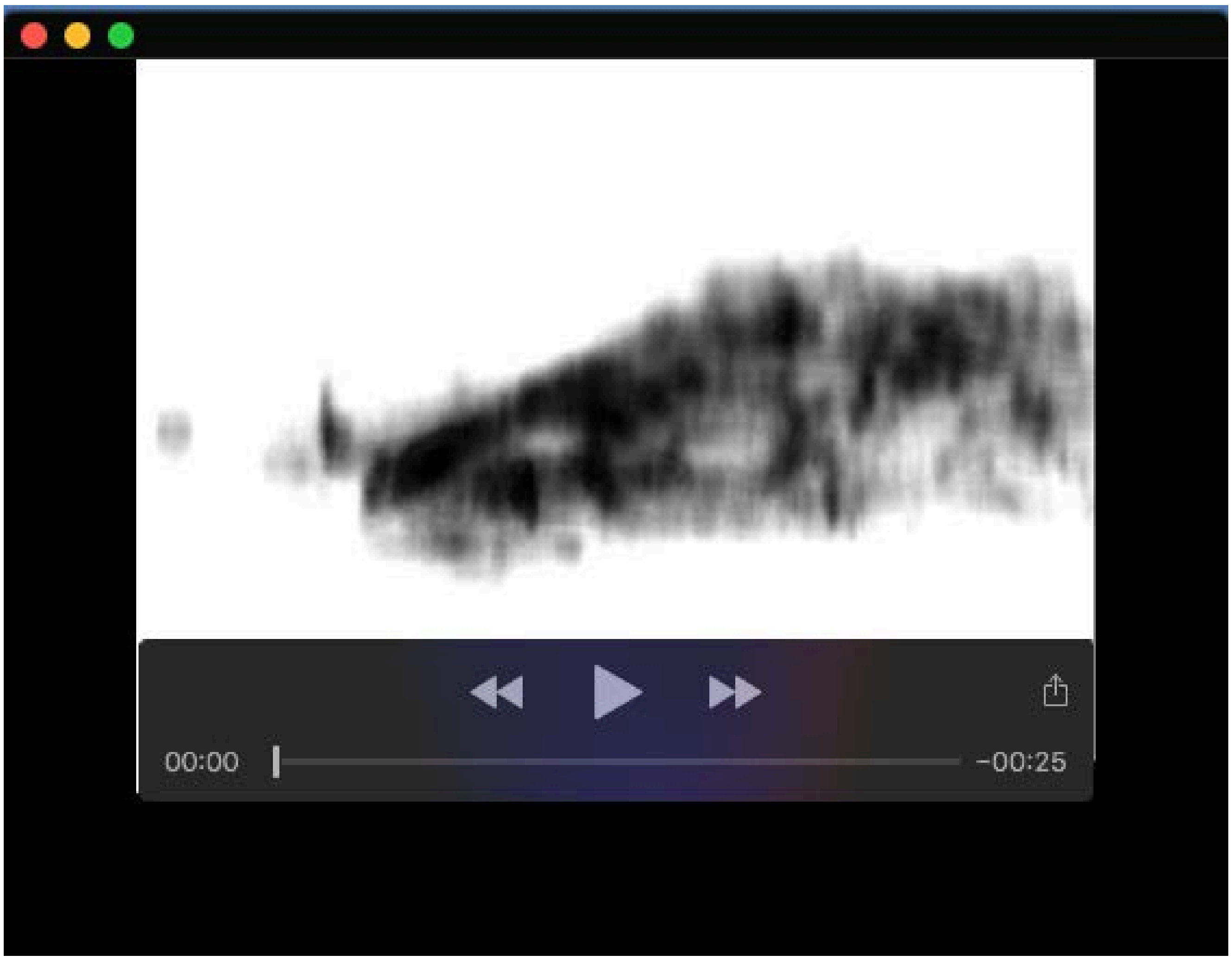

Movie 13. 3D rendition of an ESC colony plated on laminin.

360˚ rotation of a 3D rendition of the membrane staining (CellMask^{TM}, black) of a colony of ESCs plated on laminin.

Journal of Cell Science • Supplementary information

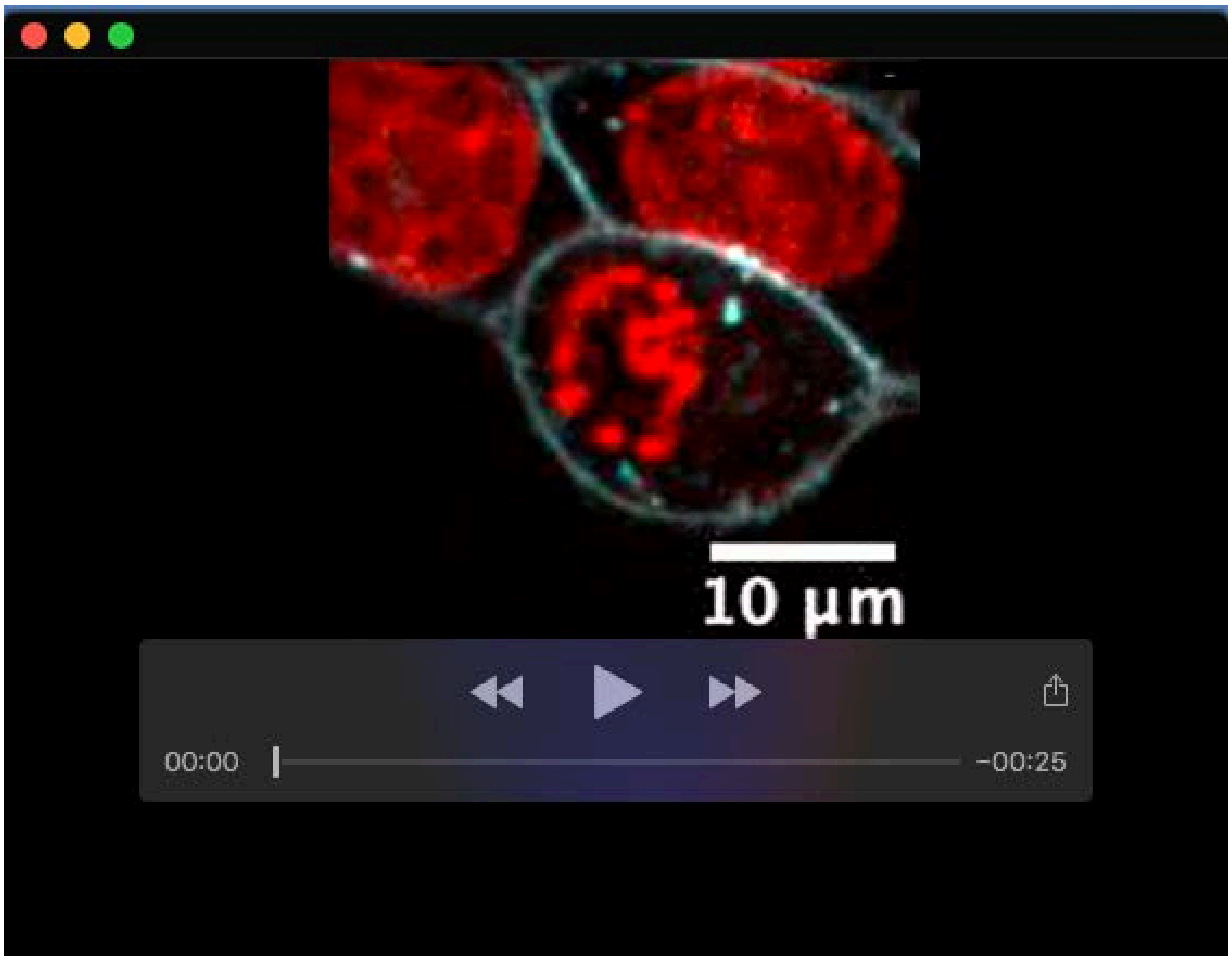

Movie 14. Example of an ESC dividing at the periphery of a 2D colony plated on laminin.

Time-lapse (spinning-disk confocal microscopy) images of a colony of ESCs expressing H2B-RFP (red) and labeled with CellMask™ deep red (cyan), plated on laminin (see Materials and Methods section) with one cell dividing at the periphery of the colony. One frame is shown every 5 min. One Z plane is shown. Scale bar: 10 µm.

Journal of Cell Science • Supplementary information
