# Supplementary material for: Three-dimensional geometry controls division symmetry in stem cell colonies
Source: J Cell Sci. 2021 Jul 29;134(14):jcs255018. doi: 10.1242/jcs.255018 (PMC8349555; doi:10.1242/jcs.255018)
Supplement: Reviewer comments [file joces-134-255018_review_history.pdf]

Journal of Cell Science

Three-dimensional geometry controls division symmetry in stem cell colonies

Agathe Chaigne, Matthew B. Smith, Rocio Lopez Cavestany, Edouard Hannezo, Kevin J. Chalut and Ewa K. Paluch
DOI: 10.1242/jcs.255018

Editor: Andrew Ewald

Review timeline

Original submission:	28 September 2020
Editorial decision:	18 November 2020
First revision received:	18 May 2021
Editorial decision:	14 June 2021
Second revision received:	15 June 2021
Accepted:	16 June 2021

Original submission

First decision letter

MS ID#: JOCES/2020/255018

MS TITLE: Three-dimensional geometry controls division symmetry in stem cell colonies

AUTHORS: Agathe Chaigne, Matthew B Smith, Rocio Lopez Cavestany, Edouard Hannezo, Kevin J Chalut, and Ewa K Paluch
ARTICLE TYPE: Research Article

We have now reached a decision on the above manuscript.

To see the reviewers' reports and a copy of this decision letter, please go to: https://submit-jcs.biologists.org and click on the 'Manuscripts with Decisions' queue in the Author Area. (Corresponding author only has access to reviews.)

As you will see, the three reviewers share considerable enthusiasm for the study and the manuscript though they raise a number of substantial criticisms that prevent me from accepting the paper at this stage. They suggest, however, that a revised version might prove acceptable, if you can address their concerns. If you think that you can deal satisfactorily with the criticisms on revision, I would be pleased to see a revised manuscript. We would then return it to the reviewers.

We are aware that you may be experiencing disruption to the normal running of your lab that makes experimental revisions challenging. If it would be helpful, we encourage you to contact us to discuss your revision in greater detail. Please send us a point-by-point response indicating where you are able to address concerns raised (either experimentally or by changes to the text) and where you will not be able to do so within the normal timeframe of a revision. We will then provide further guidance. Please also note that we are happy to extend revision timeframes as necessary.

Please ensure that you clearly highlight all changes made in the revised manuscript. Please avoid using 'Tracked changes' in Word files as these are lost in PDF conversion.

© 2021. Published by The Company of Biologists under the terms of the Creative Commons Attribution License (https://creativecommons.org/licenses/by/4.0/).

I should be grateful if you would also provide a point-by-point response detailing how you have dealt with the points raised by the reviewers in the 'Response to Reviewers' box. Please attend to all of the reviewers' comments. If you do not agree with any of their criticisms or suggestions please explain clearly why this is so.

Reviewer 1

Advance summary and potential significance to field

The manuscript by Chaigne et al examines variation in cell division behaviours in mouse embryonic stem cell colonies in 3D culture. The authors report that the position of dividing cells (interior vs at colony periphery)
results in differing E-cadherin-based restriction of spindle processes such that the geometry of resulting daughter cells is different.

Whilst the paper is light on mechanism and mostly phenomenological, it is beautifully performed and quantitated. This latter notion should outweigh the former. This well-written work that was a pleasure to review.

Comments for the author

Minor points:
Could the authors comment on what is the frequency of peripheral versus central division events?

The abstract states the following, “However, little is known about how cells control spindle positioning in more disorganized 3-dimensional (3D) environments, such as early mammalian embryos and a variety of adult tissues.” I would consider these more organised than an artificial 2D in vitro system.

On page 6, the authors state, “To track cell division dynamics, we used an ES cell line expressing histone 2B (H2B) tagged with RFP (see Methods and Figure S2A). ”

Figure S2A is a picture of a mouse.

Reviewer 2

Advance summary and potential significance to field

The manuscript by Chaigne et al. examines the orientation and symmetry of cell division in a 3-dimensional (3D) environment. Using mouse embryonic stem (ES) cell colonies as a model, the authors demonstrate that cells inside the colony divide symmetrically, whereas cells at the periphery display strong size asymmetries. These asymmetries correlate with high spindle mobility in metaphase and unequal E-cadherin distribution between daughter cells during cell division at the periphery. Furthermore, the authors show that 3D ES cells exiting naïve pluripotency divide more symmetrically, correlating this fact with a strong anaphase cortical NuMA recruitment. Finally, they find that an elongated shape of these cells at metaphase can be linked to enhanced division symmetry, indicating that anaphase cortical NuMA recruitment could be instructed by metaphase cell shape compared to what has been described in monolayers and epithelial tissues.

Authors present novel findings on cell division orientation and symmetry in 3D ES cell colonies as a model of disordered 3D environments in the early mammalian embryo, adding knowledge to what has been studied in 2D monolayer cultures and epithelial tissues. The authors extensively investigate these questions, analyzing cell size at division exit, spindle mobility, E-cadherin distribution, and NuMA positioning at the metaphase-anaphase stage. Even though a precise molecular mechanism is missing, this merit publication in JCS if several concerns, stated below, are addressed.

© 2021. Published by The Company of Biologists under the terms of the Creative Commons Attribution License (https://creativecommons.org/licenses/by/4.0/).

Comments for the author

The manuscript by Chaigne et al. examines the orientation and symmetry of cell division in a 3-dimensional (3D) environment. Using mouse embryonic stem (ES) cell colonies as a model, the authors demonstrate that cells inside the colony divide symmetrically, whereas cells at the periphery display strong size asymmetries. These asymmetries correlate with high spindle mobility in metaphase and unequal E-cadherin distribution between daughter cells during cell division at the periphery. Furthermore, the authors show that 3D ES cells exiting naïve pluripotency divide more symmetrically, correlating this fact with a strong anaphase cortical NuMA recruitment. Finally, they find that an elongated shape of these cells at metaphase can be linked to enhanced division symmetry, indicating that anaphase cortical NuMA recruitment could be instructed by metaphase cell shape compared to what has been described in monolayers and epithelial tissues.

Authors present novel findings on cell division orientation and symmetry in 3D ES cell colonies as a model of disordered 3D environments in the early mammalian embryo, adding knowledge to what has been studied in 2D monolayer cultures and epithelial tissues. The authors extensively investigate these questions, analyzing cell size at division exit, spindle mobility, E-cadherin distribution, and NuMA positioning at the metaphase-anaphase stage. Even though a precise molecular mechanism is missing, this merit publication in JCS if several concerns, stated below, are addressed.

Major points:
1-Figure1:
Figure 1B: -It might be better to create one dot plot instead of two, including all data (isolated, inside, periphery, orthoradial, and radial), adding two p-values more to the plot (isolated-orthoradial, isolated-radial). It is a way to present the data more clearly, showing that just the radial ES cells (not all the peripherical ones) display significant size asymmetries (p=0.0720 vs. p=0.4791).
-Could the authors analyze more ES cells inside the colony and include them in the dot plot? Around ten cells inside the colony have been analyzed vs. more than "80-100" peripheric cells.
Figure 1C: Although the smallest of the two daughter cells has the same probability of being positioned away from or towards the colony center…Could the authors analyze the volume of more cells in the plots showing the evolution of the volumes of daughter cells after cell division?

3-Figure 2 Size asymmetries at division correlate with high spindle mobility in metaphase:
Figure 2: Could the authors analyze if there is a delay in Spindle Assembly Checkpoint (SAC) satisfaction in peripheric cells vs. cells inside the colony?

Is there a delay in peripheric cells and takes more time to align all the chromosomes?
-Figure 2E/2F: N=2 experiments. The authors should consider including at least an N=3
-Page 10: Cell division symmetry increases during exit from naïve pluripotency: it should start with " We then sought to examine whether the levels of division" and finish with " displayed significantly more symmetric divisions compared to their naïve counterparts (Figure 4A, B). The remaining information of this paragraph should be included in the following section related to NuMA, and it is not related to the title described before.
-Figure 4:
-Figure 4C: The NuMa recruitment to the cortex and the spindle poles in Metaphase/anaphase is quantified by the authors to study enhanced division symmetry upon exit from naïve pluripotency. They specify all three components of the complex (NuMA/Ga/LGN) expressed and analyzed NuMA localization in Figure 4C. Could the localization of other complexes (LGN, for example) be analyzed in Figure 4B? Could NuMA localization can be changed in these cells in any way to verify symmetry is affected? For example, the authors might consider transfecting a dominant-negative form of NuMA?
-Figure 4E: The authors include a graph with experimental information from previous analyses made by other groups (Fig. 4E). This practice is quite unusual, and maybe they should confirm this information experimentally by qPCR analysis if they want to include it as experimental data and not just as a reference.
Minor points:
-Conclusion (page 7): "and that division asymmetries are highest for cells dividing in the surface of the colonies." In Figure 1B (right), the authors discriminate between cells dividing at the periphery

© 2021. Published by The Company of Biologists under the terms of the Creative Commons Attribution License (https://creativecommons.org/licenses/by/4.0/).

of the colony with the mitotic spindle orientated parallel (orthoradial) or perpendicular (radial). The radial cells displayed significant asymmetries between daughter cells, while the orthoradial ones are similar to isolated cells. In conclusion, the authors should be more specific, indicating that it refers to cells showing radial division orientation.
-Page 8, line 11: Are there differences in fluctuations of the metaphase plate positions between cells at the periphery with the spindle oriented perpendicular to the colony border (radial) and orthoradial? If there are not, Could it be included in the text?
-Page 9, line 22: ‘cells at the periphery of ES cell colonies on E-cadherin displayed spindles more stable than cells inside 3D colonies (Figure 3G: periphery E-cadherin green dots)
-Figure 5B and on page 12 (lines 17-21): “We found that for cells that displayed an elongated cell shape in metaphase (cell elongation > 1.2, red dots)…….spindle position may correlate better with metaphase cell shape than interphase cell shape”. Could the percentage of red dots (cell elongation >1,2) over the analyzed population be indicated in the text? Could the authors do the same for the analyzed interphase cells (>1,2)? Angle division <30.
-The authors refer to a paper from the lab in Press and from which we have no information (Chaigne et al., Page 6). Maybe this information should be shared with the reviewers.

<u>Reviewer 3</u>

Advance summary and potential significance to field

This study from the Paluch lab utilizes embryonic stem cell colonies as a model system to study division orientation and how it relates to daughter cell size asymmetries. They utilize ES cells cultured on a variety of substrates (gelatin—which mimics “3D” growth; laminin (2D); and E-cadherin), to examine how geometry affects division orientation, metaphase behavior, NuMA localization and daughter cell size. They demonstrate that in gelatin cultures, cells dividing at the periphery are more likely to have daughter cell asymmetries compared to cells in the colony center. They further show that cells which undergo “asymmetric cell divisions” are more likely to have high spindle mobility during metaphase, and non-homogeneous Ecad localization. When ES cells are cultured in differentiation medium, size asymmetries are reduced, and NuMA polarizes to the cell cortex during anaphase. The major strength of this study lies in its thorough quantitative approaches to examining cell behavior during mitosis, and the idea that polarized E-cadherin may direct asymmetric cell divisions. This study comes on the heels of a very interesting Dev Cell paper from the same group which showed that exit from pluripotency occurs after cell division and that abscission kinetics underlies differences in cell fate choices, while here they focus on earlier events in mitosis, namely metaphase and anaphase. Unfortunately, there is little attempt to unify these two papers in the discussion, or to make use of tools from the first paper (e.g. the naÃ¯ve pluripotency reporter Rex1::GFP) to directly examine how cell size asymmetry may relate to cell fate. There are also missed opportunities to attempt to unify some of the findings of this study by failing to examine E-
cadherin polarization as it relates to NuMA localization, substrate conditions and differentiation. Overall, I feel this is a very interesting study that merits publication in JCS if the authors can address these and other concerns.

Comments for the author

Major comments:
• Results, p6: “ES cells grow in 3D colonies…and are able to exit naïve pluripotency similarly to the cells at the peri-implantation blastocytst (Kalkan et al, 2017.” The cited study from the Austin Smith group as well as a very recent study from these authors (Chaigne, Dev Cell, 2020) used the Rex1::GFP reporter to visualize naïve pluripotency exit, yet there are no attempts made to use this tool to directly examine asymmetry in cell fate choices, and this study focuses purely on asymmetry in daughter cell size. This seems like a missed opportunity since they have this cell line in hand.

• Methods, p. 25, related to Fig. S1: “Shape instability assessment (Fig S1) and duration of the different phases of division were done by visual assessment.” As this is a binary assessment (shape instabilities or no shape instabilities), it is not clear how this was determined. What criteria are necessary for a shape instability to be counted? Was this analysis performed blind?

© 2021. Published by The Company of Biologists under the terms of the Creative Commons Attribution License (https://creativecommons.org/licenses/by/4.0/).

• Results, p.7: The conclusion that myosin-II is unlikely to be responsible for division asymmetries in ES cells is not justified in my opinion. The concentration of blebbistatin used (1 μM) is extremely low, orders of magnitude lower than the concentration used in other studies of its effect on spindle orientation (e.g. 50 μM in Luxenburg, Nat Cell Biol 2011). While I appreciate that high concentrations could impair mitotic progression, they have been well tolerated in other systems. Ideally, a range of drug concentrations would tested, or else knockdown of Myh9 for example, should be performed. At the very least, immunostaining for Myosin-II and phospho-MyoII should be performed to examine whether myosin levels or activity differ in divisions that occur at the colony center and periphery.

• It is argued that the ES cell colonies grown on gelatin represent a “3D” setting, yet as far as I can tell, most of the analyses—with the exception of cell size—were restricted to the xy dimension, although 2μm z-stacks were acquired. It is not clear if there is much angular movement of the metaphase plate in the xz or yz directions, or if the majority of it occurs in the xy-plane. Are these colonies more than one cell thick? If not, I would argue that these colonies are more accurately 2D clusters, and the differences between colonies grown on laminin vs gelatin are really more attributable to substrate (perhaps stiffness) rather than 2D vs 3D.

• How does plating ES cells on E-cadherin substrates or on laminin (Fig. 3) affect the distribution of Ecad compared to ES cells grown on gelatin? I assume this may lead to an equalization of Ecad levels across the cell periphery (unlike in colonies grown on gelatin, where Ecad is lower at the periphery). However, this is not directly shown in either case.

• The authors make the argument that asymmetries in Ecad levels are responsible for the size asymmetries observed at the colony periphery, since cells in the center have equal/uniform levels of Ecad. These conclusions are based on correlative rather than functional data. This could be tested directly by creating mosaic cultures of ES cells with and without Ecad knockdown. A prediction of their model is that cells in the center may undergo asymmetric size divisions if they border knockdown cells.

• Related to my first comment, in Fig. 4 the authors switch from “pluripotency sustaining medium 2i+LIF” to the differentiation-inducing medium N2B27 (2i+LIF without MEK and GSK-3 inhibitors or LIF). As I understand it, their previous study (Chaigne et al, Dev Cell, 2020) concluded that size asymmetries to not influence naïve pluripotency exit, but rather, abscission kinetics do. However, this study concludes that “cells exiting naïve pluripotency displayed significantly more symmetric divisions compared to their naïve counterparts (Figure 4A,B).” How can these apparently disparate results be rectified? Again, it would be very informative to utilize the REX1::GFP reporter in these studies to directly relate NuMA localization and cell size asymmetries to cell fate choices (e.g., naïve pluripotency exit, and whether this occurs symmetrically or asymmetrically)

• It is observed that cells with elongated axes at metaphase are more likely to occur in cells grown in differentiation medium (exiting naïve pluripotency), where NuMA becomes polarized during anaphase. Does this bear any relationship to asymmetries in E-cadherin localization? While I appreciate that the authors did grow cells on Ecad substrates and demonstrated they are more likely to have elongated shapes at metaphase, this assumes that Ecad substrates lead to more homogenous Ecad expression, which is never shown. Presumably, cells exiting pluripotency may show reduced Ecad anisotropy, which could be tested by simple immunostaining.

Minor comments:
• Figures 2 and 3 are out of order in the compiled PDF.
• Discussion, p. 14: “While many studies have investigated the control of cell division orientation and the mechanisms of spindle positioning in isolated cells or epithelia…” The authors ignore a large body of work studying division orientation in the skin epidermis, one of the better studied mammalian epithelial systems. In fact, a recent paper (Lough et al, Elife, 2019) demonstrated that adherens junction proteins play an important role in division orientation particularly in late mitosis.

© 2021. Published by The Company of Biologists under the terms of the Creative Commons Attribution License (https://creativecommons.org/licenses/by/4.0/).

First revision

Author response to reviewers' comments

Response to Reviewers' comments for Chaigne et al., "Three-dimensional geometry controls division symmetry in stem cell colonies".

We thank all Reviewers for their positive assessment of the manuscript. Below (in red) we provide a point by point response to the Reviewers' comments.

Reviewer 1 Advance Summary and Potential Significance to Field:
The manuscript by Chaigne et al examines variation in cell division behaviours in mouse embryonic stem cell colonies in 3D culture. The authors report that the position of dividing cells (interior vs at colony periphery) results in differing E-cadherin-based restriction of spindle processes such that the geometry of resulting daughter cells is different.

Whilst the paper is light on mechanism and mostly phenomenological, it is beautifully performed and quantitated. This latter notion should outweigh the former. This well-written work that was a pleasure to review.

We thank the Reviewer for their positive assessment of our work.

Reviewer 1 Comments for the Author:

Minor points:
Could the authors comment on what is the frequency of peripheral versus central division events?

We have now added a quantification of the proportion of peripheral divisions in new Figure S1E.

The abstract states the following, "However, little is known about how cells control spindle positioning in more disorganized 3-dimensional (3D) environments, such as early mammalian embryos and a variety of adult tissues." I would consider these more organised than an artificial 2D in vitro system.

We have removed the term "more disorganized", which can indeed be misleading, from the abstract.

On page 6, the authors state, "To track cell division dynamics, we used an ES cell line expressing histone 2B (H2B) tagged with RFP (see Methods and Figure S2A). " Figure S2A is a picture of a mouse.

The relevance of Figure S2A was not explained clearly enough in our original submission. This experiment is a control to test the ability of the H2B-RFP-ES cell line we use to contribute to an embryo, and thus to test the cells' stem cell potential. Figure S2A (now Figure S1C in the revised manuscript) is a picture of a chimeric mouse resulting from the injection of the H2B-RFP cells inside a blastocyst of an albino C57BL6 mouse. The albino host mouse is entirely white. The observation that the coat of the chimeric mouse displays considerable brown patches shows that the injected cells integrated well into the blastocyst and significantly contributed to the embryo and resulting mouse. We have clarified this in the text and legend.

Reviewer 2 Advance Summary and Potential Significance to Field:

The manuscript by Chaigne et al. examines the orientation and symmetry of cell division in a 3-dimensional (3D) environment. Using mouse embryonic stem (ES) cell colonies as a model, the authors demonstrate that cells inside the colony divide symmetrically, whereas cells at the periphery display strong size asymmetries. These asymmetries correlate with high spindle mobility in metaphase and unequal E-cadherin distribution between daughter cells during cell division at the periphery. Furthermore, the authors show that 3D ES cells exiting naïve

© 2021. Published by The Company of Biologists under the terms of the Creative Commons Attribution License (https://creativecommons.org/licenses/by/4.0/).

pluripotency divide more symmetrically, correlating this fact with a strong anaphase cortical NuMA recruitment. Finally, they find that an elongated shape of these cells at metaphase can be linked to enhanced division symmetry, indicating that anaphase cortical NuMA recruitment could be instructed by metaphase cell shape compared to what has been described in monolayers and epithelial tissues.

Authors present novel findings on cell division orientation and symmetry in 3D ES cell colonies as a model of disordered 3D environments in the early mammalian embryo, adding knowledge to what has been studied in 2D monolayer cultures and epithelial tissues. The authors extensively investigate these questions, analyzing cell size at division exit, spindle mobility, E-cadherin distribution, and NuMA positioning at the metaphase-anaphase stage. Even though a precise molecular mechanism is missing, this merit publication in JCS if several concerns, stated below, are addressed.

We thank the Reviewer for their positive assessment of our work.

Major points:

1-Figure1:

Figure 1B: -It might be better to create one dot plot instead of two, including all data (isolated, inside, periphery, orthoradial, and radial), adding two p-values more to the plot (isolated-orthoradial, isolated-radial). It is a way to present the data more clearly, showing that just the radial ES cells (not all the peripherical ones) display significant size asymmetries (p=0.0720 vs. p=0.4791).

We have updated this figure and now present the data in one graph. To this aim, we have removed the “periphery” group as it is the combination of the “orthoradial” and “radial” groups. This presentation indeed increases the clarity of the figure.

-Could the authors analyze more ES cells inside the colony and include them in the dot plot? Around ten cells inside the colony have been analyzed vs. more than "80-100" peripheric cells.

We have now performed more live imaging and included more cells dividing inside the colony in the graph in Figure 1B. The updated quantification includes 43 cells dividing inside the colony and 87 peripheric cells (41 dividing orthoradially and 46 dividing radially).

Figure 1C: Although the smallest of the two daughter cells has the same probability of being positioned away from or towards the colony center…Could the authors analyze the volume of more cells in the plots showing the evolution of the volumes of daughter cells after cell division?

While Figure 1C showed volume evolution for only two cells in each plot, Figure 1D reported the volume ratios of the two daughter cells 15 minutes after cytokinesis onset for cells dividing at the periphery of colonies. In the revised version, we have included the time evolution of volumes for more cells in Figure S1F, to present more specific examples. We have also indicated in Figure 1C and Figure S1F the timepoint (15 min after cytokinesis) at which the volume ratio analysis presented in Figure 1D was done. We hope that these clarifications address the concerns raised by the Reviewer.

3-Figure 2 Size asymmetries at division correlate with high spindle mobility in metaphase:

Figure 2: Could the authors analyze if there is a delay in Spindle Assembly Checkpoint (SAC) satisfaction in peripheric cells vs. cells inside the colony? Is there a delay in peripheric cells and takes more time to align all the chromosomes?

We have now measured the duration of cell division, as a proxy for SAC satisfaction. We found no difference in cell division duration between cells dividing inside and at the periphery of the colonies, suggesting no difference in SAC satisfaction timing between these two configurations. To

© 2021. Published by The Company of Biologists under the terms of the Creative Commons Attribution License (https://creativecommons.org/licenses/by/4.0/).

further test this, we also performed live imaging experiments to test the effects on division duration of SAC inhibition with the drug Reversine. Upon bypassing the SAC, we found that, as expected, the duration of cell division was shortened in both configurations, and a higher proportion of dividing cells exhibited lagging chromosomes. However, there was still no difference in division duration between cells dividing inside and at the periphery of colonies. This indicates that SAC-independent parts of cell division proceed with similar dynamics in both configurations. Together, these experiments strongly suggest that there is no delay in SAC satisfaction in peripheric cells. These new results have been added in new Figure 2J-L.

-Figure 2E/2F: N=2 experiments. The authors should consider including at least an N=3

We apologise, there was a mistake in the legends text. In these experiments, the number of experiments was in fact N=3. We have updated the legend accordingly.

-Page 10: Cell division symmetry increases during exit from naïve pluripotency: it should start with " We then sought to examine whether the levels of division" and finish with " displayed significantly more symmetric divisions compared to their naïve counterparts (Figure 4A, B). The remaining information of this paragraph should be included in the following section related to NuMA, and it is not related to the title described before.

We have revised the manuscript accordingly.

-Figure 4:
-Figure 4C: The NuMa recruitment to the cortex and the spindle poles in Metaphase/anaphase is quantified by the authors to study enhanced division symmetry upon exit from naïve pluripotency. They specify all three components of the complex (NuMA/Ga/LGN) expressed and analyzed NuMA localization in Figure 4C. Could the localization of other complexes (LGN, for example) be analyzed in Figure 4B? Could NuMA localization can be changed in these cells in any way to verify symmetry is affected? For example, the authors might consider transfecting a dominant-negative form of NuMA?

While all three components of the NuMA/Gai/LGN complex are indeed expressed in ES cells, we focused on NuMA partly for technical reasons since a good antibody is available. Furthermore, in addition to its role through the NuMA/Gai/LGN complex, NuMA can also act independently to specify spindle orientation together with the proteins 4.1G/R (Kiyomitsu and Cheeseman, 2013). 4.1G is expressed in mouse embryonic stem cells at all stages of exit from pluripotency, as well as 4.1R (albeit at lower levels at all stages) (Kalkan et al., 2017; Yang et al., 2019). Therefore, we believe NuMA is a better candidate for exploring the mechanisms of cortex polarisation than LGN or Gai. We have added this information, which we believe supports our choice to focus on NuMA, to the revised manuscript.

Regarding experiments perturbing NuMA localisation: unfortunately, NuMA performs many roles during cell division; in addition to its function in controlling spindle orientation (Bosveld et al., 2016; Kiyomitsu and Cheeseman, 2013 and many others), it is involved in spindle pole focusing (Khodjakov et al., 2003; Merdes et al., 1996; Silk et al., 2009) and nucleus reformation at the end of cell division (Compton and Cleveland, 1993; Rajeevan et al., 2020; Serra-Marques et al., 2020). Therefore, dominant negative strategies, such as the injection of a blocking antibody, lead to defects at all stages of mitosis, and in particular the formation of micronuclei (Kallajoki 1993), which could be interpreted as the result of very asymmetric divisions due to spindle mispositioning, but could also result from defects at other stages. Such confounding effects make it difficult to explore the effects of NuMA inhibition on division symmetry. siRNA against NuMA has proven difficult to use, because NuMA is a very long and abundant protein (Silk 2009); furthermore, depletion would have the same confounding effects as blocking NuMA. Blocking or depleting NuMA would thus interfere with division globally and would not allow us to test the consequences of mislocalisation of NuMA on spindle dynamics and division symmetry. Previous studies on the role of cortical NuMA in 2D tissues have thus mostly relied on physical disruption of astral microtubules that contact NuMA at the cortex using laser ablation (Bosveld et al., 2016) which is experimentally very challenging in ES cells, as they are very small compared to cells where spindle ablation has previously been performed, and form 3D colonies. Therefore, investigating the importance of cortical NuMA would require a complex experiment, involving

© 2021. Published by The Company of Biologists under the terms of the Creative Commons Attribution License (https://creativecommons.org/licenses/by/4.0/).

for example establishing optogenetic line where light would induce acute NuMA degradation or designing a construct that would lead to NuMA mislocalisation. Such tools are, to our knowledge, not available. We thus believe that an experiment changing NuMA localisation would be beyond the scope of this study at the best of times. At present, due to the COVID pandemic, we have limited access to the lab with all group members working in shifts, making performing such an experiment practically unrealistic. Instead, we now clearly explain the rationale of our focus on NuMA in the revised manuscript (p. 13) and have added a discussion of possible approaches that would allow in the future to directly test its function in ES cell division (p. 18).

-Figure 4E: The authors include a graph with experimental information from previous analyses made by other groups (Fig. 4E). This practice is quite unusual, and maybe they should confirm this information experimentally by qPCR analysis if they want to include it as experimental data and not just as a reference.

This graph was just meant to serve as further motivation to focus on NuMA. Following the Reviewer's suggestion, we have removed this figure panel and now only cite the data in the text.

Minor points:

-Conclusion (page 7): "and that division asymmetries are highest for cells dividing in the surface of the colonies." In Figure 1B (right), the authors discriminate between cells dividing at the periphery of the colony with the mitotic spindle orientated parallel (orthoradial) or perpendicular (radial). The radial cells displayed significant asymmetries between daughter cells, while the orthoradial ones are similar to isolated cells. In conclusion, the authors should be more specific, indicating that it refers to cells showing radial division orientation.

We thank the reviewer for pointing this out, and have clarified the text accordingly.

-Page 8, line 11: Are there differences in fluctuations of the metaphase plate positions between cells at the periphery with the spindle oriented perpendicular to the colony border (radial) and orthoradial? If there are not, Could it be included in the text?

We did not notice any differences in fluctuations between cells dividing radially and orthoradially, although the n number is small. We have added this in the Results text and have now highlighted cells dividing radially and orthoradially in the dot plot (Figure 2E).

-Page 9, line 22: 'cells at the periphery of ES cell colonies on E-cadherin displayed spindles more stable than cells inside 3D colonies (Figure 3G: periphery E-cadherin green dots)

We have added this clarification to the text.

-Figure 5B and on page 12 (lines 17-21): "We found that for cells that displayed an elongated cell shape in metaphase (cell elongation > 1.2, red dots).......spindle position may correlate better with metaphase cell shape than interphase cell shape". Could the percentage of red dots (cell elongation >1,2) over the analyzed population be indicated in the text? Could the authors do the same for the analyzed interphase cells (>1,2)? Angle division <30.

We have added this information to the manuscript.

-The authors refer to a paper from the lab in Press and from which we have no information (Chaigne et al., Page 6). Maybe this information should be shared with the reviewers.

The paper is now published and we have added the reference accordingly (Agathe Chaigne and others, 'Abscission Couples Cell Division to Embryonic Stem Cell Fate', Developmental Cell, 2020 https://doi.org/10.1016/j.devcel.2020.09.001).

Reviewer 3 Advance Summary and Potential Significance to Field:
This study from the Paluch lab utilizes embryonic stem cell colonies as a model system to study division orientation and how it relates to daughter cell size asymmetries. They utilize ES cells

© 2021. Published by The Company of Biologists under the terms of the Creative Commons Attribution License (https://creativecommons.org/licenses/by/4.0/).

cultured on a variety of substrates (gelatin—which mimics "3D" growth; laminin (2D); and E-cadherin), to examine how geometry affects division orientation, metaphase behavior, NuMA localization and daughter cell size. They demonstrate that in gelatin cultures, cells dividing at the periphery are more likely to have daughter cell asymmetries compared to cells in the colony center. They further show that cells which undergo "asymmetric cell divisions" are more likely to have high spindle mobility during metaphase, and non-homogeneous Ecad localization. When ES cells are cultured in differentiation medium, size asymmetries are reduced, and NuMA polarizes to the cell cortex during anaphase. The major strength of this study lies in its thorough quantitative approaches to examining cell behavior during mitosis, and the idea that polarized E-cadherin may direct asymmetric cell divisions. This study comes on the heels of a very interesting Dev Cell paper from the same group which showed that exit from pluripotency occurs after cell division and that abscission kinetics underlies differences in cell fate choices, while here they focus on earlier events in mitosis, namely metaphase and anaphase. Unfortunately, there is little attempt to unify these two papers in the discussion, or to make use of tools from the first paper (e.g. the naïve pluripotency reporter Rex1::GFP) to directly examine how cell size asymmetry may relate to cell fate. There are also missed opportunities to attempt to unify some of the findings of this study by failing to examine E- cadherin polarization as it relates to NuMA localization, substrate conditions and differentiation. Overall, I feel this is a very interesting study that merits publication in JCS if the authors can address these and other concerns.

We thank the reviewer for their positive assessment of our work. Please see below for our answers to the specific comments.

Reviewer 3 Comments for the Author:

Major comments:

- Results, p6: "ES cells grow in 3D colonies…and are able to exit naïve pluripotency similarly to the cells at the peri-implantation blastocytst (Kalkan et al, 2017." The cited study from the Austin Smith group as well as a very recent study from these authors (Chaigne, Dev Cell, 2020) used the Rex1::GFP reporter to visualize naïve pluripotency exit, yet there are no attempts made to use this tool to directly examine asymmetry in cell fate choices, and this study focuses purely on asymmetry in daughter cell size. This seems like a missed opportunity since they have this cell line in hand.

This is a very good point and we should have more clearly discussed this in the text. In fact, we have investigated whether asymmetric division affects cell fate choices in the other study mentioned by the Reviewer, Chaigne Dev Cell 2020. In that previous paper, we showed that the two daughter cells exit naïve pluripotency in a highly correlated manner, irrespective of the level of asymmetry of the division. Furthermore, inducing strong size asymmetry between daughter cells at division (by placing cells in confinement in microchannels) did not result in different timings of naïve pluripotency exit in the daughter cells (Figure 3G-H in Chaigne Dev Cell 2020). Thus, asymmetric division of naive cells does not appear to directly affect the timing of early differentiation. Instead, we speculate that heterogeneity in cell size and, as a result, in cell cycle duration, resulting from asymmetric divisions could be important for the overall dynamics of fate transitions at the population level. We had briefly discussed this point in the Discussion section of the originally submitted manuscript (paragraph starting with "Intriguingly, our recent study showed that the strong division asymmetries displayed by ES cells do not appear to affect the dynamics of naïve pluripotency exit (Chaigne *et al*).," p. 15 of the original submission) but we have realized that the conclusions of our previous paper were not discussed explicitly enough. We have now extended and clarified this discussion in the revised version (p.17 of the revised manuscript). We hope that this revised discussion helps unifying the two papers and clarifies our conclusions.

- Methods, p. 25, related to Fig. S1: "Shape instability assessment (Fig S1) and duration of the different phases of division were done by visual assessment." As this is a binary assessment (shape instabilities or no shape instabilities), it is not clear how this was determined. What criteria are necessary for a shape instability to be counted? Was this analysis performed blind?

© 2021. Published by The Company of Biologists under the terms of the Creative Commons Attribution License (https://creativecommons.org/licenses/by/4.0/).

In our original submission, this analysis had been done using a qualitative criterion and was indeed performed blind. Specifically, 3D time-lapses of cell dynamics were assessed visually, and a cell was classified as unstable when it showed extensive membrane deformations in 3D from one time point to the next, over 4 frames or more. We have now clarified this in the Methods. To address the Reviewer's comment, we have now complemented this analysis using automated quantifications of curvature to test our classification using a more objective criterion for instabilities. Specifically, we segmented the cells in the midplane in 2D and quantified the curvature at each point along the cell contour, we then calculated the variance of the curvature over the whole cell contour and computed a "cell shape variability" parameter, defined as the variance of the curvature variance over time (Figure S2B-D of the revised manuscript and details in the Methods section). We then assessed cell shape variability for the cells we had classified visually as stable or unstable (using 3D qualitative assessment). We finally performed a ROUT analysis to identify outliers that could have been misclassified. We found that there was a clear difference in cell shape variability between cells that we initially classified at stable and unstable, and that only 20 cells out of 168 needed to be removed from the analysis after this further quantitative analysis. We have decided to combine the visual assessment and the new automated analysis in the revised manuscript, because the automated analysis, while more unbiased, could only be performed in 2D whereas the visual assessment considered the 3-dimensional cell stacks. We thus believe that the combined use of both methods best addresses the concern raised by the Reviewer. We further used the cell shape variability parameter to quantitatively compare shape instabilities in control cells and in cells treated with Blebbistatin (new Figure S2J).

- Results, p.7: The conclusion that myosin-II is unlikely to be responsible for division asymmetries in ES cells is not justified in my opinion. The concentration of blebbistatin used (1 μM) is extremely low, orders of magnitude lower than the concentration used in other studies of its effect on spindle orientation (e.g. 50 μM in Luxenburg, Nat Cell Biol 2011). While I appreciate that high concentrations could impair mitotic progression, they have been well tolerated in other systems. Ideally, a range of drug concentrations would tested, or else knockdown of Myh9 for example, should be performed. At the very least, immunostaining for Myosin-II and phospho-MyoII should be performed to examine whether myosin levels or activity differ in divisions that occur at the colony center and periphery.

We absolutely agree that our experiments are not sufficient to conclude that Myosin-II is not responsible for division asymmetries. In fact, the only thing we meant to conclude in this section of the results was that Myosin-II driven polar contractions (the shape instabilities discussed in the previous point above) are not responsible for division asymmetries. We believe that our results do show this, since the low doses of blebbistatin we use are sufficient to inhibit polar shape instabilities in cells dividing at the periphery of colonies (Figure S2J), yet this treatment does not reduce division asymmetries in these cells (Figures S2K). Thus, polar contractions (which appear to be myosin driven, since low doses of blebbistatin considerably reduce them) are not likely to drive division asymmetries. We realised that this was not explained sufficiently clearly in the text and have now clarified that we do not mean to imply that Myosin-II is not involved, only that polar cortical contractions are not involved.

- It is argued that the ES cell colonies grown on gelatin represent a "3D" setting, yet as far as I can tell, most of the analyses—with the exception of cell size—were restricted to the xy dimension, although 2μm z-stacks were acquired. It is not clear if there is much angular movement of the metaphase plate in the xz or yz directions, or if the majority of it occurs in the xy-plane. Are these colonies more than one cell thick? If not, I would argue that these colonies are more accurately 2D clusters, and the differences between colonies grown on laminin vs gelatin are really more attributable to substrate (perhaps stiffness) rather than 2D vs 3D.

Naïve ES cell colonies, when grown on gelatin (the culture condition we use throughout most of the paper), are much more than one layer thick and are in fact dome shaped, as shown in Figure S1 and Movies 1-4. Naïve ES cell colonies only display a 2D cluster structure when cells are cultured on E- Cadherin or laminin (as in Figure 3C and Figures S3A, S4A). The 3D nature of ES cell colonies considerably complicates image analysis. Nonetheless, most of our analysis was performed in 3D (not only cell size (Figure 1B-D, Figure 2 F,G,I, Figure 3E, Figure S1F, Figure

© 2021. Published by The Company of Biologists under the terms of the Creative Commons Attribution License (https://creativecommons.org/licenses/by/4.0/).

S2F,K, Figure S4C,D), but also spindle angle measurements and MSD (Figure 2C-E, Figure 3G)). The only analyses performed in 2D were the comparison of cell sizes between naive cells and cells exiting naive pluripotency (Figure 4B,D) which was performed in 2D in the equatorial plane because cells exiting naive pluripotency spread on the substrate, and the comparisons of NuMA and E-Cadherin intensities at the equatorial cortex (Figure 5A,B,D-F,H). We realised that the text was not very clear on these points, and have now further highlighted the 3D nature of the ES cell colonies, and clarified which analysis was done in 2D and which in 3D in the revised manuscript.

- How does plating ES cells on E-cadherin substrates or on laminin (Fig. 3) affect the distribution of Ecad compared to ES cells grown on gelatin? I assume this may lead to an equalization of Ecad levels across the cell periphery (unlike in colonies grown on gelatin, where Ecad is lower at the periphery). However, this is not directly shown in either case.

To address this point, we have performed immunostainings of E-Cadherin in cell colonies on the different substrates (Figure S3A,B in the revised manuscript). We found that, as for cells plated on gelatin, in cells plated on laminin E-Cadherin levels are lower at the periphery of cell clusters. For cells plated on E-Cadherin, cellular E-Cadherin becomes depleted from cell-cell junctions and relocalizes to the bottom surface of the cells (see Reviewer Figure below and Figure S3). Therefore, this culture condition leads to enhanced contact with E-Cadherin at the bottom side of the cell, but not at cell-cell junctions. The aim of the experiments with cells plated on E-Cadherin in the paper was to test how exposing both future daughter cells to equal levels of E-Cadherin through contact with the E- Cadherin substrate affected cell division. Indeed, cells divide parallel to the substrate when plated on E-Cadherin, thus the 2 daughter cells are exposed to similar levels of E-Cadherin through their bottom surface. We have now clarified this in the text.

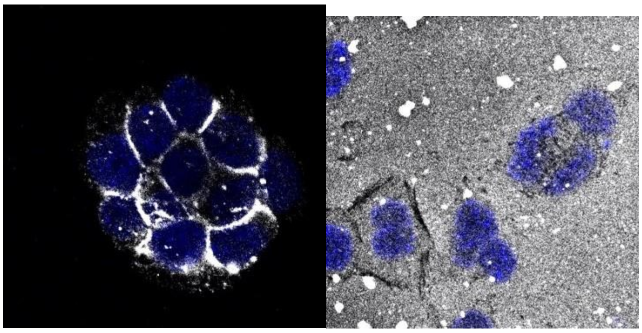

Reviewer figure: Examples of a bottom plane, close to the surface of the culture dish, of naïve ES cells plated on gelatin (left) or E-Cadherin (right) and stained with Hoechst (blue) and an E-Cadherin antibody (white). Cells plated E-Cadherin display almost no E-Cadherin at cell-cell contacts and most of the E-Cadherin localises to the bottom surface presumably to engage with the E-Cadherin on the dish. The strong background in the E-Cadherin channel results from staining of the E-Cadherin on the substrate.

- The authors make the argument that asymmetries in Ecad levels are responsible for the size asymmetries observed at the colony periphery, since cells in the center have equal/uniform levels of Ecad. These conclusions are based on correlative rather than functional data. This could be tested directly by creating mosaic cultures of ES cells with and without Ecad knockdown. A prediction of their model is that cells in the center may undergo asymmetric size divisions if they border knockdown cells.

We did attempt to test our hypothesis through the experiments with cells plated on E-Cadherin coated substrates. Indeed, we showed that plating the cells on E-Cadherin, where colonies adopt a 2D cluster structure and where, as a result, both daughter cells are always exposed to comparable levels of E- Cadherin through their contacts with the E-Cadherin coated substrate, considerably reduced cell division asymmetries. We have now clarified the motivation of this experiment in the text, also through addition of the new Figure S3 as discussed above. We agree with the Reviewer’s comment that this experiment does not provide the most direct causal demonstration. However, creating mosaic colonies with cells ko for E-Cadherin, as suggested by the Reviewer, would impair colony formation, and thus would make it impossible to assess

© 2021. Published by The Company of Biologists under the terms of the Creative Commons Attribution License (https://creativecommons.org/licenses/by/4.0/).

division asymmetries at the periphery of colonies.
Indeed, E-cadherin ko and kd ES cells have been shown to be unable to form colonies, precisely because they cannot adhere to their neighbours (Soncin et al., 2009, referenced in the manuscript). Thus, creating mosaic colonies would lead to rapid demixing of the E-Cadherin ko or kd cells. This experiment would thus not allow us to test the effects of absence of E-cadherin on division symmetry in a 3D context, since cells would not integrate into the 3D aggregates.

• Related to my first comment, in Fig. 4 the authors switch from "pluripotency sustaining medium 2i+LIF" to the differentiation-inducing medium N2B27 (2i+LIF without MEK and GSK-3 inhibitors or LIF). As I understand it, their previous study (Chaigne et al, Dev Cell, 2020) concluded that size asymmetries to not influence naïve pluripotency exit, but rather, abscission kinetics do. However, this study concludes that "cells exiting naïve pluripotency displayed significantly more symmetric divisions compared to their naïve counterparts (Figure 4A,B)." How can these apparently disparate results be rectified? Again, it would be very informative to utilize the REX1::GFP reporter in these studies to directly relate NuMA localization and cell size asymmetries to cell fate choices (e.g., naïve pluripotency exit, and whether this occurs symmetrically or asymmetrically)

We do not think that these observations are disparate. As also discussed in our response to point 1 above, in our recent study (Chaigne et al., 2020), we found that naive cells exhibit asymmetric division but that division asymmetry does not influence the dynamics of exit from naïve pluripotency (as shown by the use of the REX1::GFP reporter that the reviewer mentions). Rather, we speculate that division asymmetries may play another role, such as introducing heterogeneity in cell size and as a result, cell cycle duration, which could be important for the overall dynamics of the population fate transitions. It is indeed possible that asymmetries at division do not directly affect fate transitions, but rather result from an overall lack of regulation of cell size at cell division in ES cells.
We have expanded the discussion on this point in the revised manuscript (p. 17).

• It is observed that cells with elongated axes at metaphase are more likely to occur in cells grown in differentiation medium (exiting naïve pluripotency), where NuMA becomes polarized during anaphase. Does this bear any relationship to asymmetries in E-cadherin localization? While I appreciate that the authors did grow cells on Ecad substrates and demonstrated they are more likely to have elongated shapes at metaphase, this assumes that Ecad substrates lead to more homogenous Ecad expression, which is never shown. Presumably, cells exiting pluripotency may show reduced Ecad anisotropy, which could be tested by simple immunostaining.

As also discussed above, we have now performed immunostainings of E-Cadherin during exit from pluripotency on the different substrates and analysed the levels of E-Cadherin (Figure S3A,B). As discussed above, we found that E-Cadherin levels are very low at cell-cell junctions and the outer cortex when cells are plated on E-Cadherin, presumably because E-Cadherin relocalizes to the bottom surface of the cells to engage with the cadherin coated substrate. Interestingly, when cells exit naive pluripotency on any of the three substrates investigated, E-Cadherin localization in the cell colonies is largely unchanged, suggesting that the increased symmetry of division we observed during exit from naïve pluripotency is not primarily due to a change in cell-cell junctions. Therefore, the changes in metaphase cell shape and NuMA localization are unlikely to be driven by changes in E-Cadherin localization. Instead, we believe that E-Cadherin asymmetries lead to division asymmetries in naïve cells and that enhanced division symmetry during early differentiation of ES cells is controlled by different mechanisms. We have clarified this in the revised Discussion and have also added new Figure S3 to the manuscript.

Minor comments:
• Figures 2 and 3 are out of order in the compiled PDF.

• We are sorry for this confusion, we will correct this.

• Discussion, p. 14: "While many studies have investigated the control of cell division orientation and the mechanisms of spindle positioning in isolated cells or epithelia…" The authors ignore a large body of work studying division orientation in the skin epidermis, one of the better studied mammalian epithelial systems. In fact, a recent paper (Lough et al, Elife, 2019) demonstrated that

© 2021. Published by The Company of Biologists under the terms of the Creative Commons Attribution License (https://creativecommons.org/licenses/by/4.0/).

adherens junction proteins play an important role in division orientation, particularly in late mitosis.

We apologise for missing this; we have updated the references and Discussion accordingly.

References

Bosveld, F., Markova, O., Guirao, B., Martin, C., Wang, Z., Pierre, A., Balakireva, M., Gaugue, I., Ainslie, A., Christophorou, N., et al. (2016). Epithelial tricellular junctions act as interphase cell shape sensors to orient mitosis. Nature *530*, 495–498.

Chaigne, A., Labouesse, C., White, I.J., Agnew, M., Hannezo, E., Chalut, K.J., and Paluch, E.K. (2020). Abscission Couples Cell Division to Embryonic Stem Cell Fate. Developmental Cell.

Compton, D.A., and Cleveland, D.W. (1993). NuMA is required for the proper completion of mitosis. The Journal of Cell Biology *120*, 947–957.

Kalkan, T., Olova, N., Roode, M., Mulas, C., Lee, H.J., Nett, I., Marks, H., Walker, R., Stunnenberg, H.G., Lilley, K.S., et al. (2017). Tracking the embryonic stem cell transition from ground state pluripotency. Development *144*, 1221–1234.

Khodjakov, A., Copenagle, L., Gordon, M.B., Compton, D.A., and Kapoor, T.M. (2003). Minus-end capture of preformed kinetochore fibers contributes to spindle morphogenesis. Journal of Cell Biology *160*, 671–683.

Kiyomitsu, T., and Cheeseman, I.M. (2013). Cortical Dynein and Asymmetric Membrane Elongation Coordinately Position the Spindle in Anaphase. Cell *154*, 391–402.

Merdes, A., Ramyar, K., Vechio, J.D., and Cleveland, D.W. (1996). A Complex of NuMA and Cytoplasmic Dynein Is Essential for Mitotic Spindle Assembly. Cell *87*, 447–458.

Rajeevan, A., Keshri, R., Kapoor, S., and Kotak, S. (2020). NuMA interaction with chromatin is vital for proper chromosome decondensation at the mitotic exit. Mol Biol Cell *31*, 2437–2451.

Serra-Marques, A., Houtekamer, R., Hintzen, D., Canty, J.T., Yildiz, A., and Dumont, S. (2020). The mitotic protein NuMA plays a spindle-independent role in nuclear formation and mechanics. J Cell Biol *219*.
Silk, A.D., Holland, A.J., and Cleveland, D.W. (2009). Requirements for NuMA in maintenance and establishment of mammalian spindle poles. Journal of Cell Biology *184*, 677–690.

Soncin, F., Mohamet, L., Eckardt, D., Ritson, S., Eastham, A.M., Bobola, N., Russell, A., Davies, S., Kemler, R., Merry, C.L.R., et al. (2009). Abrogation of E-Cadherin-Mediated Cell-Cell Contact in Mouse Embryonic Stem Cells Results in Reversible LIF-Independent Self-Renewal: E-Cadherin is Required for LIF-Dependent Self-Renewal. STEM CELLS *27*, 2069–2080.

Yang, P., Humphrey, S.J., Cinghu, S., Pathania, R., Oldfield, A.J., Kumar, D., Perera, D., Yang, J.Y.H., James, D.E., Mann, M., et al. (2019). Multi-omic Profiling Reveals Dynamics of the Phased Progression of Pluripotency. Cell Systems *8*, 427-445.e10.

Second decision letter

MS ID#: JOCES/2020/255018

MS TITLE: Three-dimensional geometry controls division symmetry in stem cell colonies

© 2021. Published by The Company of Biologists under the terms of the Creative Commons Attribution License (https://creativecommons.org/licenses/by/4.0/).

AUTHORS: Agathe Chaigne, Matthew B Smith, Rocio Lopez Cavestany, Edouard Hannezo, Kevin J Chalut, and Ewa K Paluch
ARTICLE TYPE: Research Article

We have now reached a decision on the above manuscript.

To see the reviewers' reports and a copy of this decision letter, please go to: https://submit-jcs.biologists.org and click on the 'Manuscripts with Decisions' queue in the Author Area. (Corresponding author only has access to reviews.)

As you will see, the reviewers gave favourable reports and made one suggestion in terms of including xy and xz views. I hope that you will be able to make this change because I would like to be able to accept your paper. Please explain any edits you make in the cover letter. I will review the revised manuscript myself.

We are aware that you may be experiencing disruption to the normal running of your lab that makes experimental revisions challenging. If it would be helpful, we encourage you to contact us to discuss your revision in greater detail. Please send us a point-by-point response indicating where you are able to address concerns raised (either experimentally or by changes to the text) and where you will not be able to do so within the normal timeframe of a revision. We will then provide further guidance. Please also note that we are happy to extend revision timeframes as necessary.

Please ensure that you clearly highlight all changes made in the revised manuscript. Please avoid using 'Tracked changes' in Word files as these are lost in PDF conversion.

I should be grateful if you would also provide a point-by-point response detailing how you have dealt with the points raised by the reviewers in the 'Response to Reviewers' box. Please attend to all of the reviewers' comments. If you do not agree with any of their criticisms or suggestions please explain clearly why this is so.

<u>Reviewer 1</u>

Advance summary and potential significance to field

The authors have addressed all of my (previously minor) comments. This revision was similarly a lovely paper to read and review.

Comments for the author

No further revisions required.

<u>Reviewer 2</u>

Advance summary and potential significance to field

The manuscript by Chaigne et al. examines the orientation and symmetry of cell division in a 3-dimensional (3D) environment. Using mouse embryonic stem (ES) cell colonies as a model, the authors demonstrate that cells inside the colony divide symmetrically, whereas cells at the periphery display strong size asymmetries. These asymmetries correlate with high spindle mobility in metaphase and unequal E-cadherin distribution between daughter cells during cell division at the periphery. Furthermore, the authors show that 3D ES cells exiting naïve pluripotency divide more symmetrically, correlating this fact with a strong anaphase cortical NuMA recruitment. Finally, they find that an elongated shape of these cells at metaphase can be linked to enhanced division symmetry, indicating that anaphase cortical NuMA recruitment could be instructed by metaphase cell shape compared to what has been described in monolayers and epithelial tissues.

Comments for the author

© 2021. Published by The Company of Biologists under the terms of the Creative Commons Attribution License (https://creativecommons.org/licenses/by/4.0/).

The manuscript by Chaigne et al. increases our understanding of cell division behaviour in mouse embryonic stem cell colonies in 3D culture. In the revised manuscript, the authors' updated figure 1, which now looks more intuitive. They increased the clarity of figure 1B showing one plot instead of two, and performing more live-cell imaging experiments from cells dividing inside the colony. The authors have also updated figure 1C and S1F, including some details to clarify the data shown. They have performed additional experiments to show there is no delay in SAC-satisfaction in peripheric cells. As a result, the data have been included in a new figure 2J-L. The authors analyzed the NuMa recruitment to the cortex and the spindle poles to study enhanced division symmetry upon exit from naïve pluripotency in the first manuscript. Although many experiments have been done to disrupt NuMA localization, they very well explain the difficulties of the multiple existing approaches. In conclusion, the authors have addressed the major points suggested by this reviewer, revised and clarified the manuscript as requested. I believe it is now ready for publication in J Cell Science".

Reviewer 3

Advance summary and potential significance to field

This revised manuscript from Chaigne et al thoroughly addresses each of my comments—as well as those of the other two reviewers—and is a further improvement on an already strong and comprehensive study. In particular, they have done a very nice job clarifying portions of the text that I found vague or ambiguous, so that it now reads quite well. My one minor comment is that it is difficult to appreciate the differences between the 3D (gelatin) and 2D (laminin) cultures unless one looks at the Supplementary Movies. My suggestion is to add xz and/or yz slices to some of their images to further impress this important point.

Comments for the author

Minor comment:
• The addition of xz and/or yz views of the 3D colonies to the examples shown in Fig 1A and Fig S1A/B (like the views shown in Supplementary Movie 1) would be extremely helpful in terms of conveying the 3D structure of these cultures. This is not obvious from the xy slices and stacks shown in the current figures.

Second revision

Author response to reviewers' comments

To better show the 3D structures of the colonies, we have added side views of 3D renditions of cells plated on gelatin, laminin and E-Cadherin.

Third decision letter

MS ID#: JOCES/2020/255018

MS TITLE: Three-dimensional geometry controls division symmetry in stem cell colonies

AUTHORS: Agathe Chaigne, Matthew B Smith, Rocio Lopez Cavestany, Edouard Hannezo, Kevin J Chalut, and Ewa K Paluch
ARTICLE TYPE: Research Article

I am happy to tell you that your manuscript has been accepted for publication in Journal of Cell Science, pending standard ethics checks.

© 2021. Published by The Company of Biologists under the terms of the Creative Commons Attribution License (https://creativecommons.org/licenses/by/4.0/).
